# Senolytics decrease senescent cells in humans: Preliminary report from a clinical trial of Dasatinib plus Quercetin in individuals with diabetic kidney disease

**DOI:** 10.1016/j.ebiom.2019.08.069

**Published:** 2019-09-18

**Authors:** LaTonya J. Hickson, Larissa G.P. Langhi Prata, Shane A. Bobart, Tamara K. Evans, Nino Giorgadze, Shahrukh K. Hashmi, Sandra M. Herrmann, Michael D. Jensen, Qingyi Jia, Kyra L. Jordan, Todd A. Kellogg, Sundeep Khosla, Daniel M. Koerber, Anthony B. Lagnado, Donna K. Lawson, Nathan K. LeBrasseur, Lilach O. Lerman, Kathleen M. McDonald, Travis J. McKenzie, João F. Passos, Robert J. Pignolo, Tamar Pirtskhalava, Ishran M. Saadiq, Kalli K. Schaefer, Stephen C. Textor, Stella G. Victorelli, Tammie L. Volkman, Ailing Xue, Mark A. Wentworth, Erin O. Wissler Gerdes, Yi Zhu, Tamara Tchkonia, James L. Kirkland

**Affiliations:** a Cellular Senescence and Translation and Pharmacology Programs, Robert and Arlene Kogod Center on Aging, Mayo Clinic, United States of America; b Division of Geriatric Medicine and Gerontology, Department of Medicine, Mayo Clinic, United States of America; c Division of Nephrology and Hypertension, Department of Medicine, Mayo Clinic, United States of America; d Department of Medicine Clinical Trials Unit, Department of Medicine, Mayo Clinic, United States of America; e Division of Hematology, Department of Medicine, Mayo Clinic, United States of America; f Division of Endocrinology, Department of Medicine, Mayo Clinic, United States of America; g Department of Surgery, Mayo Clinic, United States of America; h Department of Physiology and Biomedical Engineering, Mayo Clinic, United States of America; i Division of Hospital Medicine, Department of Medicine, Mayo Clinic, United States of America; j Department of Physiology, Mayo Clinic, United States of America; k Department of Physical Medicine and Rehabilitation, Mayo Clinic, United States of America; l Office of Research Regulatory Support, Mayo Clinic, United States of America; m Division of General Internal Medicine, Department of Medicine, Mayo Clinic, United States of America

**Keywords:** Senolytics, Cellular senescence, Dasatinib, Quercetin, Diabetic kidney disease, Senescence-associated secretory phenotype

## Abstract

**Background:**

Senescent cells, which can release factors that cause inflammation and dysfunction, the senescence-associated secretory phenotype (SASP), accumulate with ageing and at etiological sites in multiple chronic diseases. Senolytics, including the combination of Dasatinib and Quercetin (D + Q), selectively eliminate senescent cells by transiently disabling pro-survival networks that defend them against their own apoptotic environment. In the first clinical trial of senolytics, D + Q improved physical function in patients with idiopathic pulmonary fibrosis (IPF), a fatal senescence-associated disease, but to date, no peer-reviewed study has directly demonstrated that senolytics decrease senescent cells in humans.

**Methods:**

In an open label Phase 1 pilot study, we administered 3 days of oral D 100 mg and Q 1000 mg to subjects with diabetic kidney disease (*N* = 9; 68·7 ± 3·1 years old; 2 female; BMI:33·9 ± 2·3 kg/m^2^; eGFR:27·0 ± 2·1 mL/min/1·73m^2^). Adipose tissue, skin biopsies, and blood were collected before and 11 days after completing senolytic treatment. Senescent cell and macrophage/Langerhans cell markers and circulating SASP factors were assayed.

**Findings:**

D + Q reduced adipose tissue senescent cell burden within 11 days, with decreases in p16^INK4A^-and p21^CIP1^-expressing cells, cells with senescence-associated β-galactosidase activity, and adipocyte progenitors with limited replicative potential. Adipose tissue macrophages, which are attracted, anchored, and activated by senescent cells, and crown-like structures were decreased. Skin epidermal p16^INK4A+^ and p21^CIP1+^ cells were reduced, as were circulating SASP factors, including IL-1α, IL-6, and MMPs-9 and −12.

**Interpretation:**

“Hit-and-run” treatment with senolytics, which in the case of D + Q have elimination half-lives <11 h, significantly decreases senescent cell burden in humans.

**Fund:**

NIH and Foundations.

ClinicalTrials.gov Identifier: NCT02848131. Senescence, Frailty, and Mesenchymal Stem Cell Functionality in Chronic Kidney Disease: Effect of Senolytic Agents.

**Research in context**

**Evidence before this study**

Senescent cells accumulate in tissues with ageing and at etiological sites in multiple chronic diseases, including adipose tissue in diabetes. Senescent cells can release products that cause inflammation and death of non-senescent cells, the senescence-associated secretory phenotype (SASP). Intermittent administration of the senolytic drug combination, Dasatinib plus Quercetin (D + Q), which transiently disables the pro-survival pathways that defend senescent cells against their own apoptotic environment, selectively eliminates senescent cells from mouse and human cell cultures, ageing mice, mice with insulin resistance and many other chronic disorders, and freshly-isolated adipose tissue explants from obese, diabetic human subjects. In the first clinical trial of senolytics, D + Q alleviated physical dysfunction in patients with idiopathic pulmonary fibrosis (IPF), a progressive, fatal, cellular senescence-associated disease. In another clinical trial, prolonged D administration to patients with systemic sclerosis appeared to reduce the SASP and other senescence markers in skin biopsies. To date, no peer-reviewed clinical trial report has directly demonstrated that administering senolytics decreases senescent cells.

**Added value of this study**

Here, in an open-label Phase 1 pilot study, we show for the first time that senolytic drugs decrease senescent cell abundance in humans. A 3-day oral course of D + Q in subjects with diabetic kidney disease (DKD) reduced adipose tissue senescent cell burden 11 days later, as indicated by decreases in cells with markers of senescence: p16^INK4A^-and p21^CIP1^-expressing cells, cells with senescence-associated β-galactosidase (SAβgal) activity, and adipocyte progenitors with limited replicative potential. Consistent with decreased overall senescent cell burden in humans treated with senolytics, skin epidermal p16^INK4A^- and p21^CIP1^-expressing cells were also reduced, as were key SASP factors, including interleukin (IL)-1α, IL-6, and MMPs 9 & 12, in blood. Thus, “hit-and-run” treatment with senolytic agents, which in the case of D + Q have elimination half-lives of <11 h, is sufficient to decrease senescent cell burden in humans. Thereafter, senescent cell burden remains low for days to weeks, consistent with the >2 weeks it takes for new senescent cells to develop (at least in cell culture).

**Implications of all the available evidence**

Interventions targeting fundamental ageing processes such as cellular senescence could delay, prevent, or alleviate multiple age-related diseases and disorders as a group, instead of one-at-a-time, as per the Geroscience Hypothesis. Increasingly in mice, this hypothesis appears to be true. Combined with the first clinical trial of senolytic agents showing that D + Q improves physical function in patients with IPF published earlier this year in this journal, our current article showing that D + Q actually decreases senescent cell burden in humans is consistent with the possibility that the Geroscience Hypothesis may also hold true for humans. If clinical trials over the next few years support and extend our findings to show that these agents can alleviate additional age- and cellular senescence-related disorders and diseases (beyond IPF) and reduce senescent cell burden (beyond adipose tissue and skin and as reflected by decreased SASP factors in blood), senolytics might become an entirely new path for alleviating currently untreatable chronic diseases and enhancing human healthspan.

Alt-text: Unlabelled Box

## 1 Introduction

Cellular senescence is a cell fate that entails essentially irreversible replicative arrest and that was first reported in serially sub-cultured human cells in 1961 [1]. Insults such as serial passaging, DNA damage, exposure to the damage-associated molecular pattern molecules that accumulate in injured or chronically-diseased tissues, and metabolic insults can cause cells to become senescent [2]. Senescent cells and pre-senescent cells with limited replicative potential accumulate with ageing in multiple tissues in which they are linked to functional declines, including in adipose tissue, where abundance of adipocyte progenitors with decreased proliferative capacity increases with ageing [[3], [4], [5], [6], [7], [8], [9], [10], [11], [12], [13], [14], [15]]. Even in younger mice or human subjects, senescent cell abundance is increased in adipose tissue in conditions such as obesity associated with metabolic dysfunction as well as in chronic kidney disease [[15], [16], [17], [18], [19], [20]]. Indeed, senescent cells accumulate at etiologic sites of multiple chronic diseases across the age range [2,5,[8], [9], [10],^12^,^16^,^17^,[21], [22], [23], [24], [25]]. Senescent cells are metabolically active and can acquire a pro-inflammatory, tissue-destructive, pro-apoptotic senescence-associated secretory phenotype (SASP) [26], such that transplanting even small numbers of senescent mouse or human adipocyte progenitors or senescent autologous ear fibroblasts into younger mice can result in local [27] and systemic disability and disease [28], yet senescent cells themselves are resistant to apoptosis [29].

Senolytics are drugs that specifically target senescent cells by inducing apoptosis of senescent but not non-senescent cells [30]. Work that led to the discovery of senolytics was prompted by a 2004 article showing that the age-related increase in senescent cells is delayed by interventions that increase healthspan: caloric restriction or the Prop-1 deficiency in long-lived Ames mice [31]. This suggested to us that interventions to reduce senescent cell burden might extend healthspan and alleviate age-related diseases. Of note, our efforts to discover senolytics began before and progressed independently from devising and making transgenic mice from which highly p16^Ink4a^-expressing cells, some of which are senescent, can be eliminated by activating the product of the transgene, an approach that is not translatable to humans. Senolytics act by facilitating apoptosis of senescent cells due to their own SASP, not by directly targeting all cells that have high p16^Ink4a^ expression.

By definition, the target of senolytics is senescent cells, not a molecule or a single biochemical pathway. The first senolytic drugs, Dasatinib (D) and Quercetin (Q), were discovered using a mechanism-based approach instead of the random high-throughput screening usually used for drug discovery [32]. Through a bioinformatics strategy that leveraged proteomic and transcriptomic data, we discovered networks of senescent cell anti-apoptotic pathways (SCAPs) that enable senescent cells to survive despite their own SASP. We verified this role of these networks in senescent cells by using RNA interference to show that targeting SCAP nodes, such as BCL-xL in the case of human umbilical vein endothelial cells, kills senescent cells. Using a priori knowledge about their molecular targets and mechanisms of action, D and Q were identified as being potentially senolytic based upon their predicted ability to transiently disable these SCAP networks [32]. The senolytic activity of D and Q was first verified in human senescent vs. non-senescent cells. We found that different types of senescent cells use different SCAPs or even redundant combinations of SCAPs to evade apoptosis, meaning that agents targeting a single SCAP would only be expected to eliminate a subset of senescent cells. Indeed, we found that D, which was selected based on the specific tyrosine kinases and other key SCAP elements that it targets, is senolytic in the case of human adipocyte progenitors, as predicted from the particular SCAPs these cells depend on. We found that the flavonoid Q, which was specifically selected because it targets BCL-2 family members as well as HIF-1α and particular nodes in PI3-kinase and p21-related anti-apoptotic pathways, is senolytic in the case of human endothelial cells, again as predicted. Q does not target senescent human adipocyte progenitors efficiently and D does not target senescent human endothelial cells. To extend the range of senescent cells targeted, in subsequent mouse in vivo studies and in the clinical trial reported here, we used the combination of D + Q.

Because senescent cells can take weeks to months to develop and do not divide, and because even eliminating only 30% of senescent cells can be sufficient to alleviate dysfunction in preclinical studies [3,5,7,9,23,24,28,[32], [33], [34], [35], [36], [37]], D + Q is as effective in mice if administered intermittently, for example every 2 weeks to a month, as continuously, even though D and Q have elimination half-lives of only 4 and 11 h, respectively [38,39]. This is consistent with the point that, since the target of senolytics is senescent cells, these drugs do not need to be continuously present in the circulation in the same way as drugs whose mechanism of action is to occupy a receptor, modulate an enzyme, or act on a particular biochemical pathway, at least in mice. Intermittently administering D + Q effectively circumvents any potential off-target effects due to continuous receptor occupancy or modulation of an enzyme or biochemical pathway.

Based on the many promising studies of D + Q in mice [3,5,7,9,23,24,28,[32], [33], [34], [35], [36], [37]], the experience gained from the use of D in humans for over 20 years, and the fact that Q is a natural product present in many foods such as apples, we initiated clinical trials of these agents. The first-in-human clinical trial of senolytics was a brief course of D + Q for patients with IPF, which resulted in statistically significant improvements in physical function in 14 subjects with this relentlessly progressive, debilitating, and ultimately fatal cellular senescence-driven disease, as published in January 2019 [40]. Although alleviation of this cellular senescence-related phenotype was demonstrated in that pioneering trial in tandem with trends for decreased SASP factors, and others found decreased SASP factors in a trial of continuous D administration in the skin of subjects with systemic sclerosis [41], so far, there has been no direct demonstration of senescent cell clearance by senolytic drugs in peer-reviewed published human clinical trials.

To test whether intermittent D + Q is effective in targeting senescent cells in humans, we administered a single 3 day course of oral D + Q and assayed senescent cell abundance 11 days after the last dose in subjects with DKD, the most common cause of end-stage kidney failure and which is characterized by increased senescent cell burden [18,[42], [43], [44]]. We found D + Q alleviates insulin resistance, proteinuria, and renal podocyte dysfunction caused by high fat diet-induced or genetic obesity in mice [23]. We also found that even Q alone can prevent high fat diet-induced increases in markers of senescence, renal fibrosis, decreases in renal oxygenation, and increased creatinine in mice, although Q alone did not prevent insulin resistance [20]. Diabetes and chronic kidney disease (CKD) in humans therefore represent conditions that may benefit from D + Q therapy-induced alleviation of tissue dysfunction and disease progression, which is being tested as the clinical trial reported here continues. In this interim report of findings from that trial, we found the single brief course of D + Q attenuated adipose tissue and skin senescent cell burden, decreased resulting adipose tissue macrophage accumulation, enhanced adipocyte progenitor replicative potential, and reduced key circulating SASP factors.

## 2 Materials and methods

### 2.1 Study participants and senolytic administration

Study participants were accrued from November 2016 to April 2019 at Mayo Clinic in Rochester, Minnesota. Table 1 shows the demographic and clinical characteristics of these participants. Adults aged 50–80 years with diabetes mellitus (on anti-diabetes therapy) and CKD (estimated glomerular filtration rate range 15–45 mL/min/1·73m^2^) were included. Individuals with QTc prolongation (>450 msec), liver dysfunction, non-diabetic kidney disease, dialysis, malignancy, active infection, morbid obesity (body mass index [BMI] >50 kg/m^2^), solid organ transplantation, or taking drugs known to interact with D + Q were excluded. Such drugs include those with a narrow therapeutic range that are substrates for CYP3A4, CYP2C8, CYP2C9, CYP2D6, CYP2C19, CYP1A2, or OATP1B1 or strong inhibitors or inducers of CYP3A4 (e.g., cyclosporine, tacrolimus, or sirolimus). Informed consent was obtained from study participants. The Mayo Clinic Institutional Review Board approved this study, which is registered at ClinicalTrials.gov (NCT02848131) and which is an ongoing clinical trial to determine the effectiveness of senolytics for restoring cellular, tissue, and clinical function in patients with diabetic chronic kidney disease. A single 3-day oral treatment regimen with D 100 mg daily and Q 1000 mg total daily (500 mg twice daily) was administered to all participants on Study Days 1 to 3.

**Table 1:** Baseline demographic and clinical variables of diabetic kidney disease participants treated with a Single 3-Day Oral Course of D + Q.

Variable	Participants Treated with D + Q (N = 9^a^)
Age, years	68·7 (1·0)
Male sex	7 (77·8%)
White race	8 (88·9%)
BMI, kg/m^2^	33·9 (0·8)
eGFR, mL/min/1·73m^2^	27·0 (0·7)
Diabetes Insulin therapy alone	5/9
Oral glucose lowering therapy alone	2/9
Both insulin and oral glucose lowering therapy	2/9

Data represent Mean (SEM) or Number (%).

BMI: body mass index; eGFR: estimated glomerular filtration rate.

a Adipose tissue sufficient for isolating adipose progenitors but not for immunohistochemistry or SAβgal activity or skin assays was available from 2 additional subjects (ages 70 & 75 years, BMI 31·1 & 29·4 kg/m^2^, eGFR 38 & 29 mL/min/1·73m^2^, males, white, diabetic on glucose lowering therapy alone).

### 2.2 Adipose tissue and skin sampling and plasma collection

To avoid sun-exposed areas of skin and to simultaneously obtain abdominal subcutaneous adipose tissue superficial to Scarpa's fascia, elliptical incisional biopsies of adipose tissue and attached skin were acquired from at a point to the right or left of, and 2–5 cm inferior to the navel. Abdominal subcutaneous adipose tissue (0·5–2 g) and attached skin were collected under sterile conditions through a 3–5 cm incision on Study Days 0 and 14. The site for the post-treatment biopsy was contralateral to that for the pre-treatment biopsy, with both the pre- and post-treatment biopsies being from the same dermatome. Skin attached to the adipose tissue biopsy was cut from the underlying adipose tissue. Also on Study Days 0 and 14, blood was collected. Blood samples were stored at −80 °C for subsequent analyses.

### 2.3 Immunohistochemistry, imaging of biopsies, and counting

Adipose tissue and skin biopsies were fixed in formalin and paraffin-embedded. Five μm sections were placed on slides and immunohistochemically stained with CINtec Histology monoclonal mouse anti-human p16^INK4A^ (clone E6H4; Roche Tissue Diagnostics, Indianapolis, IN), p21^CIP1^ (DCS-60·2; Cell Marque Tissue Diagnostics, Rocklin, CA), CD68 (Clone PG-M1; Dako, Denmark), and CD1a (Clone MTB1; Novocastra, Leica Biosystems, UK) antibodies using a VENTANA Discovery ULTRA instrument (Ventana Medical Systems, Oro Valley, AZ) following instructions and using materials suggested by the manufacturer. Adipose tissue and skin biopsy slides were scanned using a Motic Slide Scanner (Motic Company, China) and a 40× objective. In adipose tissue biopsies, images scanned for p16^INK4A^ and p21^CIP1^ were sliced (virtually) using Adobe Photoshop (Adobe Inc., San José, CA) to generate fields that were 400 μm × 300 μm. Thirty fields were selected for analysis from each image using a random number generator. p16^INK4A+^ and p21^CIP1+^ cells and adipocytes were counted by ImageJ [45,46]. For macrophages, an Olympus BX43 light microscope (Olympus, Japan) with a 40× objective was used to image 10 random fields, which were manually counted for CD68^+^ macrophages, crown-like structures, and adipocyte shells using the software, AMCounter (Biomedical Imaging Resource, Mayo Clinic, Rochester, MN) [47]. For skin biopsies, cells positive for p16^INK4A^, p21^CIP1^ (present in the basal layer), CD1a, and CD68 were counted and divided by the total length of the epidermis (mm) using ImageJ. All image analyses were conducted by observers who were blinded as to whether samples were pre- or post-treatment biopsies.

### 2.4 Senescence-associated β-galactosidase (SAβgal) activity assay

Adipose biopsies were washed 3 times with PBS, fixed for 5 min in PBS containing 2% (vol/vol) formaldehyde (Sigma-Aldrich, St. Louis, MO) and 0·25% glutaraldehyde (Sigma-Aldrich), and washed with PBS 3 times. Tissues were incubated in SAβgal activity solution, pH 6·0, at 37 °C for 16–18 h (overnight), washed, stained with Hoechst dye for 10 min for counting nuclei, and kept in PBS until being imaged with a fluorescence microscope (Nikon Eclipse Ti, Japan). Ten to 15 random fields were imaged per sample. SAβgal^+^ cells were determined as a function of all nuclei in the fields.

To test consistency of assays of cells with high SAβgal activity between adipose tissue biopsies, SAβgal^+^ cells were quantified as above from adipose tissue biopsies collected 15 days apart from 15 obese, premenopausal women who were not taking senolytics and who were enrolled in a separate IRB-approved study (Suppl. Table 1).

### 2.5 Blood SASP factor analyses

Plasma cytokines and MMPs were quantified using a multiplex fluorescent bead assay (Human Cytokine 42-Plex Discovery Assay and Human MMP & TIMP Discovery Assay; Eve Technologies; Calgary, Alberta, Canada).

### 2.6 Adipocyte progenitor replication assay

Adipocyte progenitors, also termed adipose tissue-derived mesenchymal stem cells or preadipocytes, were harvested from abdominal subcutaneous fat samples as previously described [48,49]. Adipose biopsies were minced and digested in 2% collagenase type I at 37 °C for 45 min (Gibco, Waltham, MA), filtered through a 100 μm cell strainer (BD Biosciences, San José, CA) to remove remaining tissue pieces, and centrifuged to pellet cells. Cells were cultured in advanced minimum-essential-medium (Thermo Fisher Scientific, Waltham, MA) supplemented with 5% platelet lysate (PLTMax, Mill Creek Life Sciences, Rochester, MN) and 2 mM l-glutamine (Invitrogen, Carlsbad, CA) in a 37 °C/5% CO_2_ incubator for 3–4 days. When 60–80% confluent, cells were passaged using TrypLE (Trypsin-like Enzyme, Invitrogen, Invitrogen, Waltham, MA). The third passage cells were collected and kept in Gibco Cell Culture Freezing Medium (Life Technologies, Carlsbad, CA) at −80 °C. Change in adipocyte progenitor numbers over time was measured in passage 3–4 cultures using a tetrazolium compound (3-[4,5-dimethylthiazol-2-yl]-5-[3-carboxymethoxyphenyl]-2-[4-sulfophenyl]-2H-tetrazolium, inner salt; MTS) assay (CellTiter 96 Non-Radioactive Cell Proliferation Assay; Promega, Madison, WI) according to the manufacturer's instructions.

### 2.7 Statistical methods

Prior to pilot study initiation, sample size estimates were determined. Pilot trial sample size estimation was based on a sample size of 9, which has 80% power to detect a difference in means of 5% (for the senescence marker, SAβgal activity), assuming a standard deviation of differences of 4·0, using a *t*-test with a 0·05 two-sided significance level. Data are presented as mean ± SE or raw values (tables and text). Welsh's unpaired t-test for unequal variances was used for comparing Day 0 vs. Day 14. The α-level for significance was 0·05. Analyses were conducted using GraphPad Prism 7·02 (San Diego, CA, USA).

## 3 Results

**Fig. 1:**
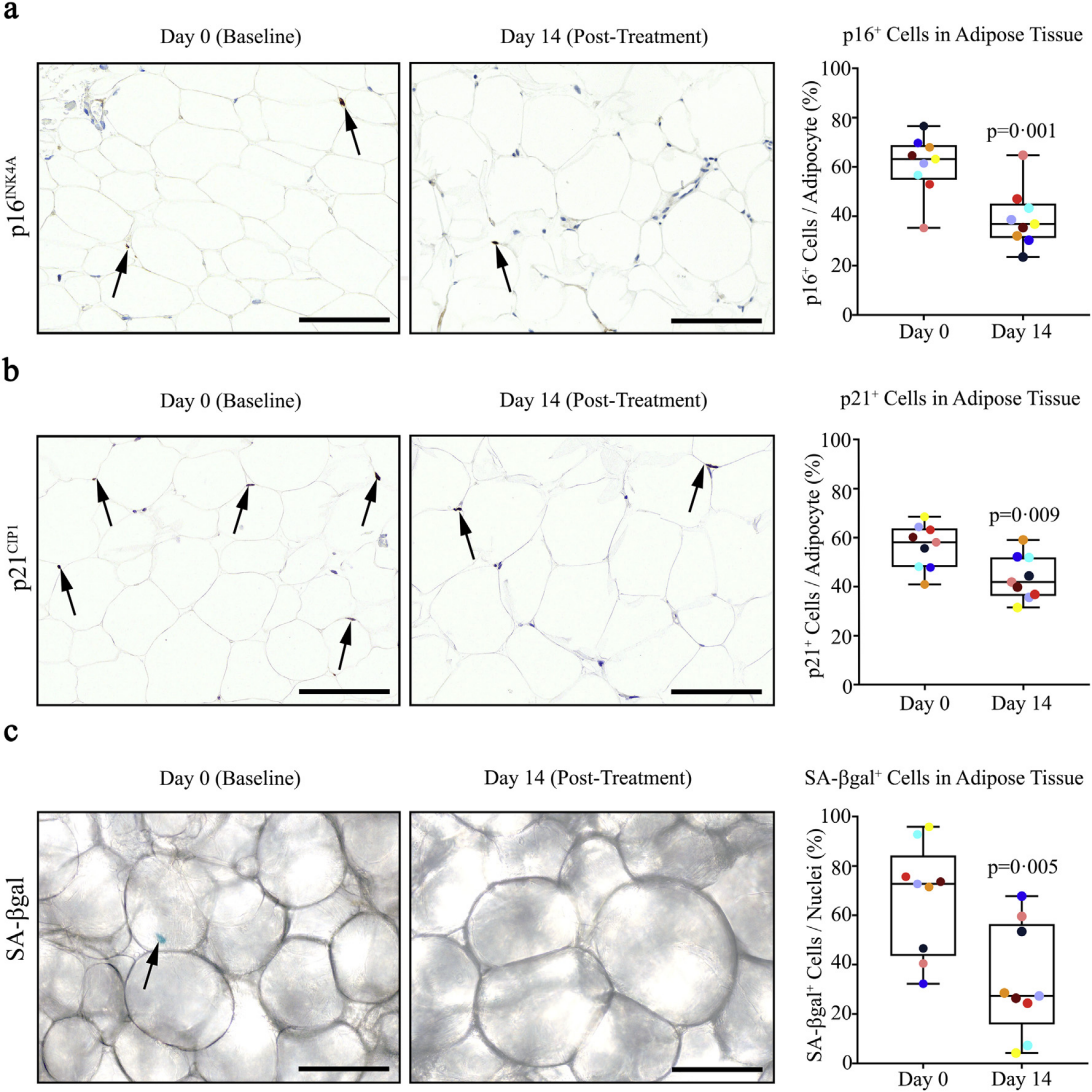
D + Q decreases human adipose tissue senescent cells. (a). D + Q significantly reduced (*p* = 0·001) abdominal subcutaneous adipose tissue p16^INK4A+^ cells. Raw values were reduced by 35% in sections of adipose tissue biopsied at Day 14 (11 days after the last dose of a 3-day course of the senolytics) vs. at baseline (Day 0). At Day 0, there were 3·18 ± 0·64 p16^INK4A+^ cells/100 adipocytes (means ± SEM in 30 fields [400 × 300 μm] at 40× magnification). Means, standard errors, and standard deviations are shown in these “box and whisker” plots. The y axis shows p16^INK4A+^ cells in the 2 biopsies from each subject at Days 0 and 14 as % of each other (Arbitrary Units). *N* = 9 subjects; Welch's unpaired 2-tailed *t*-test for unequal variances. Representative images at Days 0 and 14 are shown. (b). D + Q significantly reduced (p = 0·009) adipose tissue p21^CIP1+^ cells. Raw values were decreased 17% by 11 days after completing D + Q treatment. At baseline (Day 0), there were 3·82 ± 0·65 p21^CIP1+^ cells/100 adipocytes (*N* = 9 subjects; means ± SEM). Representative images are shown. (c). D + Q significantly reduced (p = 0·005) adipose tissue SA-βgal-expressing cells. Raw values were decreased by 62% by 11 days after completing D + Q treatment. At baseline (Day 0), there were 8·76 ± 2·51 SAβgal^+^ cells/100 nuclei (*N* = 9 subjects; mean ± SEM). Representative images are shown. Scale bars = 100 μm. Exact *p* values are indicated. Colours indicate each individual's values on Days 0 and 14.

Abdominal subcutaneous excisional adipose tissue biopsies were acquired from 9 subjects (*N* = 9; mean age: 69, range 55–79 years; 7 males and 2 females) with drug-controlled diabetes and CKD (Table 1) before and 11 days after completion of a single 3-day course of oral D + Q (D: 100 mg/day, Q: 500 mg twice daily). During the course of this study, no serious adverse events (i.e. hospitalization, kidney injury requiring dialysis, or death) occurred and no subjects required drug discontinuation. Expression of p16^INK4A^ and p21^CIP1^ were both used as markers of senescent cells, as was SAβgal activity [50,51]. D + Q significantly reduced (*p* = 0·001) adipose tissue p16^INK4A+^ cells. Raw values were reduced by 35% (Fig. 1a). Senolytics significantly reduced (*p* = 0·009) adipose tissue p21^CIP1+^ cells, with raw values being decreased by 17% (Fig. 1b). D + Q also reduced (*p* = 0·005) SAβgal^+^ cells; raw values were reduced by 62% (Fig. 1c). To exclude the possibility that SAβgal^+^ cells could have been induced in the second biopsy due to trauma from the first, we assayed adipose tissue biopsies obtained 15 days apart from women with obesity who were not treated with senolytics. SAβgal^+^ cell counts were similar in the two biopsies (Suppl. Table 1), implying the observed declines in SAβgal^+^ cells after senolytic treatment were not due to an artifact caused by serial sampling.

**Fig. 2:**
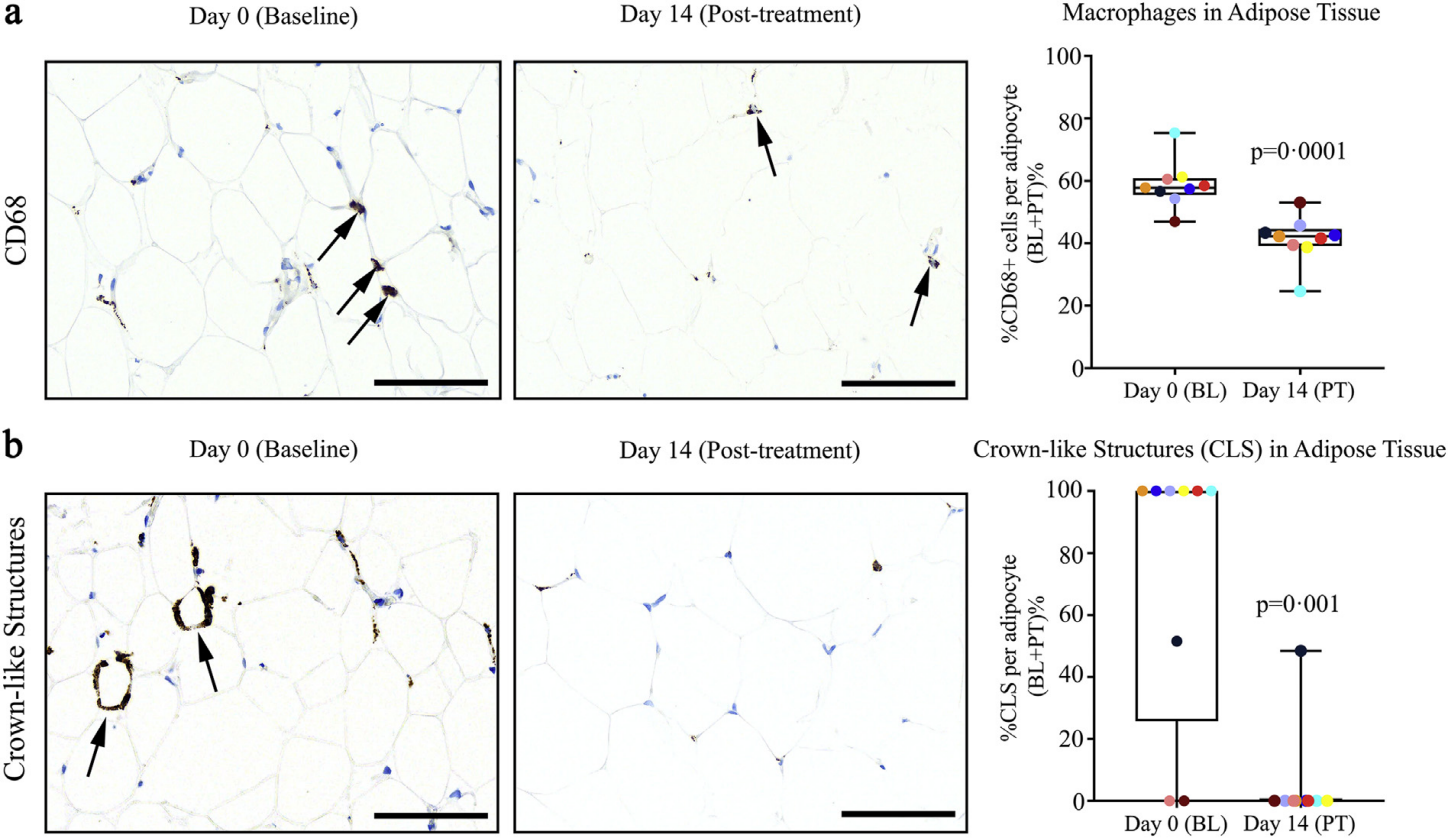
D + Q decreases human adipose tissue macrophages and crown-like structures. (a). D + Q significantly reduced (*p* = 0·0001) adipose tissue CD68^+^ macrophages relative to adipocytes. Raw numbers were decreased 28% by 11 days after completing D + Q treatment. At baseline, there were 8·4 ± 0·58 CD68^+^ macrophages/100 adipocytes (*N* = 9 subjects; mean ± SEM). Representative images at Days 0 and 14 are shown. (b). D + Q significantly reduced (*p* = 0·001) adipose tissue crown-like structures. Raw values were decreased by 11 days after D + Q treatment. At baseline (Day 0), there were 0·27 ± 0·05 CD68^+^ crown-like structures/100 adipocytes (*N* = 9 subjects; mean ± SEM). Representative images are shown. Scale bars = 100 μm. Exact *p* values are indicated. Colours indicate each individual's values on Days 0 and 14.

Senescent cells have been shown to attract, activate, and anchor macrophages in adipose tissue, so removing senescent cells should be associated with a decline in macrophage abundance [23]. To test this in humans, CD68^+^ cells were counted in the adipose tissue biopsies before vs. 11 days after completion of D + Q treatment (Fig. 2a). There were fewer CD68^+^ macrophages after treatment (*p* = 0·0001). D + Q lowered the raw numbers of macrophages per adipocyte by 28%. Senescent cells and macrophages contribute to formation of the “crown-like structures” (CLS) that are characteristic of adipose tissue in diabetes and obesity [52]. We reported that there are decreased numbers of CLS independently from any changes in body weight or food intake following intermittent senolytic treatment of diet-induced obese mice, as well as in response to genetic clearance of p16^Ink4a+^ cells [23]. Consistent with the decreases in senescent cell burden and coupled decreases in adipose tissue macrophages from humans with diabetes and CKD reported here, we found that D + Q significantly decreases CLS in abdominal subcutaneous adipose tissue (*p* = 0·001). The raw number of CLS per adipocyte decreased by 86% (Fig. 2b).

**Fig. 3:**
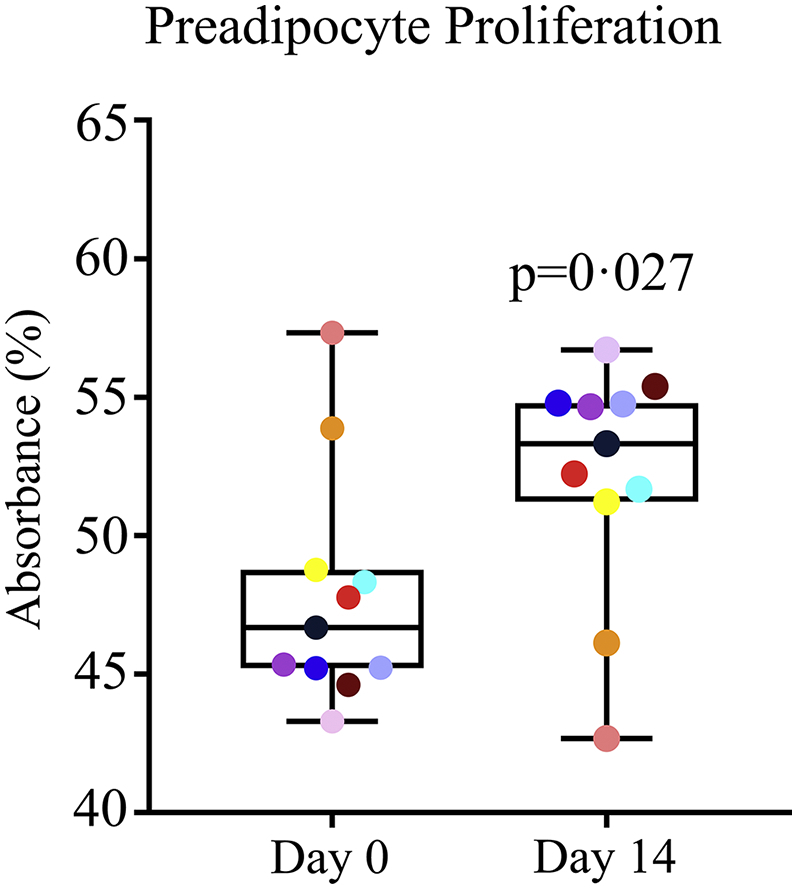
Increases in adipocyte progenitor cell density over time are enhanced following administration of D + Q, consistent with removal of cells with limited replicative potential (senescent and pre-senescent cells). Cell density/time was assayed by tetrazolium uptake in adipocyte progenitors isolated from adipose biopsies acquired before (Day 0) and 14 days after the first dose of the 3-day course of D + Q (Day 14) and cultured in parallel for 3 passages. Increases in cell density/time in adipocyte progenitors isolated after senolytic treatment were 8% greater than in adipocyte progenitors isolated before treatment (*N* = 11 subjects; Table 1). Exact p value is indicated. Colours indicate each individual's values on Days 0 and 14.

Senescent and pre-senescent cells have no or limited replicative potential, respectively, resulting in increased population doubling times as senescent and pre-senescent cells accumulate in serially-passaged cultures [1,53]. This parallels the reduced replicative potential observed in primary cell types, including skin fibroblasts and adipocyte progenitors, isolated from older mice, rats, or humans than cells isolated from younger individuals [4,14,54]. To determine if depletion of senescent adipose progenitors contributed (beyond any potential role of p16^INK4A+^ and SAβgal-expressing macrophages) to the decreases in p16^INK4A+^ and SAβgal-expressing cells in adipose tissue after senolytic treatment, we assayed replicative potential of primary adipocyte progenitors isolated from adipose tissue biopsies from the 9 subjects plus an additional 2 subjects (from whom fat was available for these analyses but not immunohistochemistry; Table 1). The adipocyte progenitors were first cultured under conditions that exclude macrophages for 3 passages [[55], [56], [57]]. Following administration of D + Q to the subjects, increases in numbers of primary adipocyte progenitors over time in cultures derived from their abdominal subcutaneous fat biopsies were greater than in the baseline cultures isolated from the biopsies before treatment, consistent with effects of removing senescent and pre-senescent cells (Fig. 3).

**Fig. 4:**
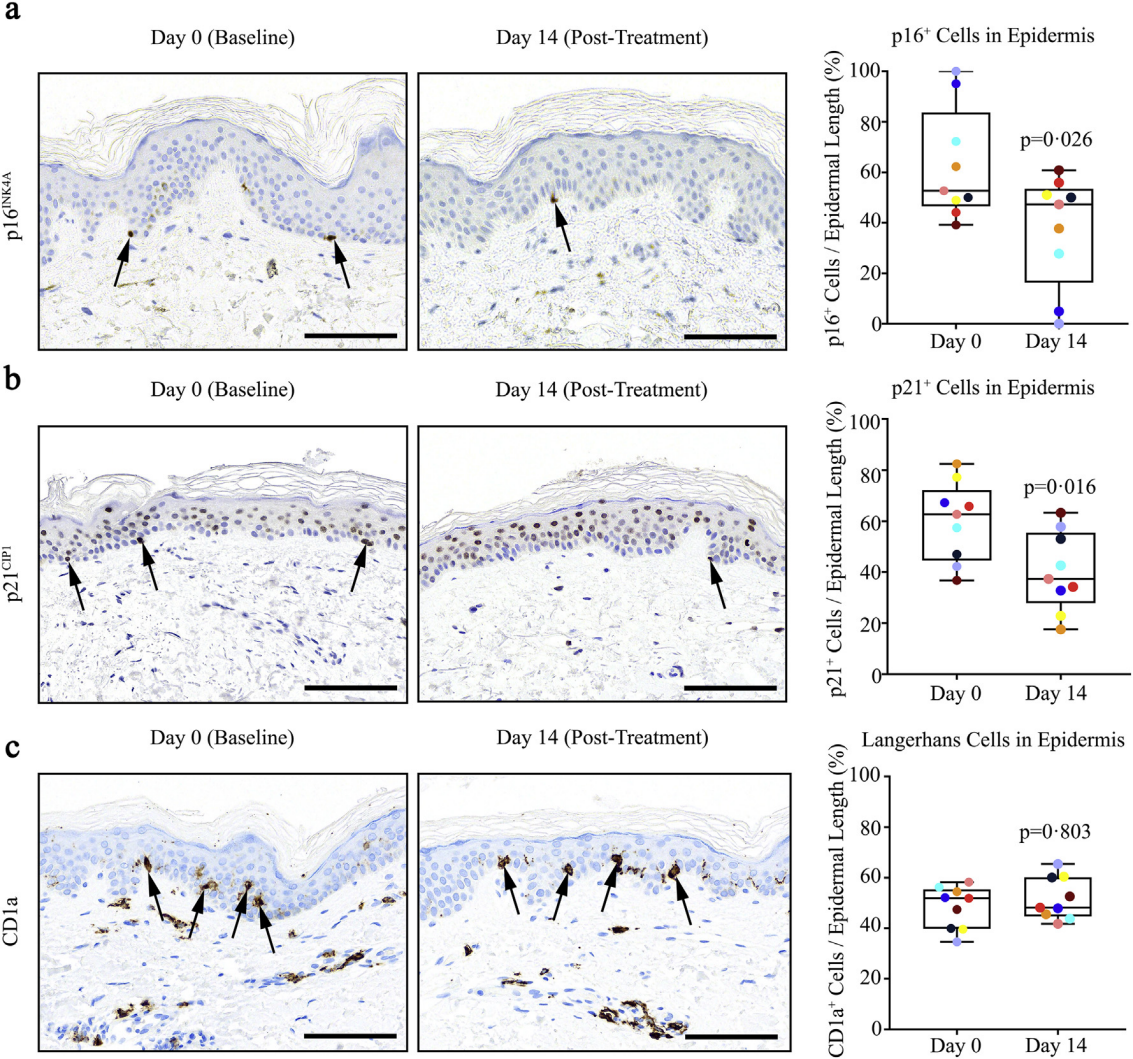
D + Q decreases human epidermal senescent cells. (a). D + Q significantly reduced (p = 0·026) human epidermal basal layer p16^INK4A+^ cells. Raw values were decreased 20% by 11 days after completing D + Q treatment. At baseline (Day 0), there were 1·95 ± 0·63 p16^INK4A+^ cells/mm of epidermis (*N* = 9 subjects; mean ± SEM). Representative images at Days 0 and 14 are shown. (b). D + Q significantly reduced (p = 0·016) human epidermal basal layer p21^CIP1+^ cells. Raw values were decreased 31% by 11 days after completing D + Q treatment. At baseline (Day 0), there were 1·71 ± 0·31 p21^CIP1+^ cells/mm of epidermis (mean ± SEM). Representative images are shown. (c). D + Q did not substantially change (*p* = .803) antigen-presenting CD1a^+^ epidermal Langerhans immune cells. At baseline (Day 0), there were 14·55 ± 2·16 CD1a^+^ cells/mm of epidermis (mean ± SEM; *N* = 9). Scale bars = 100 μm. Exact p values are indicated. Colours indicate each individual's values on Days 0 and 14.

To test if D + Q reduces senescent cell burden in tissues in addition to adipose tissue in subjects with diabetes and CKD, we analyzed the epidermal layer of skin overlying the abdominal subcutaneous adipose tissue taken before and after senolytic treatment. Similar to adipose tissue, p16^INK4A+^ and p21^CIP1+^ cells in the epidermis decreased after treatment (*p* = 0·026 and *p* = 0·016, respectively). p16^INK4A+^ cells as a function of epidermal length were 20% less abundant 11 days after completing 3 days of treatment with D + Q than at baseline (Fig. 4a) and p21^CIP1+^ cells were 31% less abundant (Fig. 4b). With regard to epidermal immune cells, Langerhans cells, which are the antigen-presenting macrophage-like resident cells in the epidermis and express CD1a [58], CD1a did not significantly decrease after D + Q treatment (*p* = .8; Fig. 4c). Resident macrophages expressing CD68 have not been reported in the epidermis. Consistent with this, while present in dermis, cells expressing CD68 were not detectible in the epidermis either before or after D + Q treatment (*N* = 9 subjects; 18 biopsies; full length of epidermis scanned with a 40× objective). Thus, the decrease in epidermal p16^INK4A+^ cells after D + Q treatment is not readily explained by a decrease in CD68^+^;p16^INK4A+^ macrophages or in the related Langerhans cells.

**Fig. 5:**
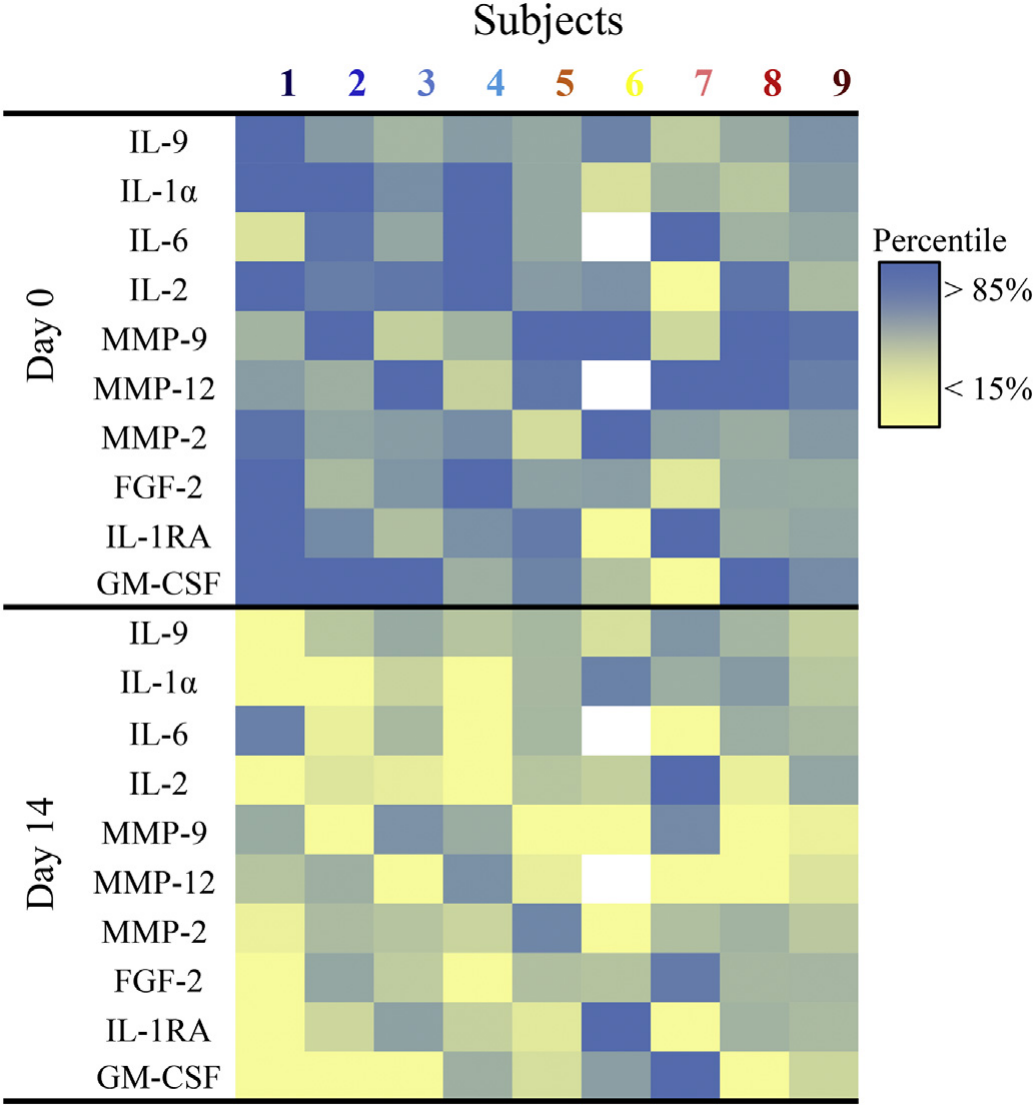
D + Q decreases plasma SASP factors. Plasma SASP factors were assayed at baseline (Day 0) and after treatment (Day 14). Key SASP factors that were significantly decreased (*p* < 0·05) after treatment are shown. Colours indicate each individual's values on Days 0 and 14.

We tested if key circulating SASP factors were decreased by the short course of senolytics. Plasma IL-1α, −2, −6, and − 9 and Matrix Metalloproteinases (MMP)-2, −9, and −12 were significantly lower 11 days after than before the 3 days of D + Q administration (Fig. 5) and IL1-RA, Fibroblast Growth Factor (FGF)-2, and Granulocyte Macrophage Colony-Stimulating Factor (GM-CSF) tended to be lower (all *p* = 0·06). Abundance of adipose tissue p16^INK4A+^ cells (relative to adipocytes) correlated significantly with plasma IL-6, IL-9, IL-1α, IL1-RA, MMP12, MMP9, and MMP-2 (R^2^ = 0·34*, 0·25*, 0·56***, 0·24*, 0·51**, 0·36**, and 0·25*, respectively; * *p* < 0·05, ** *p* < 0·005, and ***p < 0·001). These circulating SASP factor data are consistent with a systemic decrease in senescent cell abundance that remained apparent well beyond the 4 to 11 h elimination half-lives of D + Q.

## 4 Discussion

The intended target of senolytics is senescent cells. We present evidence showing that the drugs, D + Q, which are senolytic in mice and cell or tissue culture models, decrease senescent cells in humans. No individual markers of senescent cells appear to be fully sensitive and specific. Furthermore, individual factors associated with the SASP can be produced by non-senescent cells, such as immune cells. We therefore assayed several key cellular senescence markers and circulating SASP factors in tandem as well as markers of macrophages, which can exhibit features similar to those of senescent cells [59]. Macrophages are attracted, activated into a senescent-like state, and anchored by senescent cells in adipose tissue [23].

Key markers of senescent cell burden were decreased in adipose tissue and skin biopsied from subjects 11 days after completing the 3-day course of D + Q, as were key circulating SASP factors, compared to before administration of these senolytic drugs. Changes in the adipose tissue biopsies consistent with decreased senescent cells included decreases in the percent of cells with high p16^INK4A^ and p21^CIP1^ expression (Figs. 1a & b, respectively) and SAβgal activity (Fig. 1c), decreased abundance of macrophages and crown-like structures (Figs. 2a & b), and increased cell numbers over time in primary cultures of adipocyte progenitors derived from the subjects' adipose tissue biopsies (Fig. 3). The decrease in cells with high SAβgal activity was not an artifact caused by repeating biopsies: cellular SAβgal activity was consistent in repeated biopsies from obese subjects not treated with senolytics (Suppl. Table 1). D + Q decreased p16^INK4A+^ and p21^CIP1+^ cells in another tissue, the epidermal layer of skin (Figs. 4a & b, respectively). Epidermal but not dermal p16^INK4A+^ cells have been associated with cardiovascular disease risk and “biological ageing” [60]. In the epidermal layer, the decreases in p16^INK4A+^ and p21^CIP1+^ cells were independent from changes in CD1a^+^ Langerhans cells [58]. Also, the changes in skin, together with the decreases in senescent cells in adipose tissue and circulating SASP factors, suggest that oral administration of D + Q decreases overall senescent cell burden, as opposed to targeting senescent cells within a single organ or structure, such as may be the case following local injection of agents into knees or eyes.

Macrophages expressing the marker, CD68, were decreased in adipose tissue biopsies after D + Q administration vs. at baseline (Fig. 2a). This finding was expected to occur in concert with decreases in senescent cells, because senescent cells anchor macrophages within adipose tissue [23]. Part of the decrease in p16^INK4A+^ cells and cells with SAβgal activity could have been due to the decreased presence of activated macrophages, in addition to reductions in senescent cell abundance. However, it is unlikely that activated macrophages were directly eliminated by D + Q, since we previously reported that, under conditions in which D + Q caused death of senescent human adipocyte progenitors, p16^INK4A^-expressing activated macrophages were unaffected [28]. Therefore, the reduction in adipose tissue macrophage abundance we found here after subjects had completed treatment with D + Q was more likely due to the decreased macrophage attraction and anchoring within adipose tissue consequent to removing senescent cells. Consistent with this scenario, we previously reported that pre-treating obese mice with D + Q attenuates trafficking into adipose tissue of labelled monocytes injected into their tail veins compared to mice pre-treated with vehicle instead of D + Q [23]. In the mice that had not been pre-treated with senolytics, the injected labelled monocytes rapidly moved into their adipose tissue. This effect of senolytics on reducing attraction of circulating monocytes/macrophages into tissues may be a reason we found little effect of D + Q on epidermal Langerhans cells, which are essentially resident macrophages [58]. Furthermore, there were few or no CD68^+^ cells in the epidermis before or after D + Q treatment, indicating that the decline in epidermal p16^INK4A+^ cells after D + Q was not because of an effect on CD68-expressing macrophages. These points are additional evidence that direct, upstream effects of D + Q might be principally on senescent cells, with effects on immune cell types, such as adipose tissue macrophages, being a consequence of these decreases in senescent cells.

Potential of adipocyte progenitors isolated from biopsies obtained 11 days after completion of senolytic treatment and passaged three times to increase in numbers over time was increased compared to progenitors from the baseline biopsies from the same subjects (Fig. 3). These cells were cultured under conditions to exclude macrophages. Like the decreases in the percent of cells with high p16^INK4A^ expression and SAβgal activity after D + Q noted above, this is consistent with a decrease in senescent and pre-senescent cell burden. Not only is loss of replicative potential a key feature of senescent cells, those cells approaching replicative senescence have a decrease in remaining capacity to increase in numbers and, similarly, cells isolated from tissues of older subjects with more senescent cells exhibit reduced replicative potential compared to cells isolated from younger subjects [3,53,54]. Thus, our finding that potential of adipocyte progenitors cultured from adipose tissue biopsies to increase in numbers over time is enhanced by brief D + Q treatment of diabetic subjects with a high burden of senescent cells, compared to the pre-treatment baseline biopsies from the same subjects, is consistent with a direct senolytic effect of D + Q in humans.

Also consistent with D + Q causing a decline in senescent cell burden in humans, plasma levels of key SASP factors including IL-1α, −2, −6, and −9 and MMP-2, −9, and −12, were reduced after D + Q treatment (Fig. 5). The findings that the decreases in adipose tissue and skin senescent cell abundance and the decreases in systemic SASP factors were evident 11 days after the last of 3 daily oral doses of D + Q is indicative of a sustained effect of these drugs after they were no longer present in the body. This is consistent with D + Q having caused senescent cell removal. Indeed, as we reported in old mice treated for age-related osteoporosis with D + Q [5], these findings suggest senolytics may be as effective if given intermittently as continuously, despite the elimination half-lives of D and Q being <11 h. Senescent cells take weeks to over a month to form and acquire a SASP, at least in culture, meaning that senolytic agents can be administered in a “hit-and-run” fashion. This finding of a sustained decline in senescent cell burden means that D + Q is not likely to exert its effects through off-target mechanisms of the type involving continuous occupancy of a receptor or from modulating an enzyme, but rather it is more likely that D + Q kills slowly-reappearing, non-dividing senescent cells. Such intermittent administration of senolytics could reduce side effects. For example, D is usually administered continuously with a tolerable side effect profile at 100 mg/day (the dose used here for only a total of 3 days) for years or even over a decade in patients with chronic leukemias [61]. Most potential side-effects of D can be avoided though intermittent administration and, generally, serious side effects of D reverse upon temporary discontinuation [62,63], increasing the value of the opportunity to administer D intermittently, yet still benefit from its senolytic effects.

Our findings provide support for the likelihood that the beneficial effects of intermittent D + Q on cellular senescence-related phenotypes in IPF, which we reported in the first clinical trial of senolytics [40], were indeed due to reduction in senescent cell burden. Over and above the trends we noted for circulating SASP factors to be lower after than before D + Q in that first clinical trial, in the second clinical trial to examine senolytics, others documented a decrease of 53 out of 66 SASP factors, as well as decreases in a DNA damage/telomere stress-induced senescence gene set. These changes were found in comparing skin biopsies from a subset of patients with systemic sclerosis who had an initially high senescent cell burden before being treated with D (no Q) continuously for ~6 months vs. after treatment [41]. Now, in the third peer-reviewed clinical trial of senolytics reported here, we provide direct evidence for a decrease in both adipose tissue and skin epidermal senescent cell abundance and circulating SASP factors 11 days following a single 3-day course of D + Q.

Much remains to be done to optimize senolytic regimens and to identify the possibly broad range of effects and side effects of these agents in humans. If senolytic agents can be shown to be effective for several individual age-related conditions, they may prove to have a role beyond alleviating single diseases: they may be effective in reducing the multimorbidity common in elderly patients. In the first demonstration that healthspan can be improved by removing senescent cells from naturally-aged mice, we found that clearing senescent cells with D + Q improves cardiac function and vascular reactivity in old mice, alleviates frailty and increases intervertebral disc proteoglycans in progeroid mice, and enhances treadmill endurance in single-leg radiation-injured mice in early 2015 [32]. Confirming and extending that first study, we and others subsequently showed that D + Q alleviated a range of additional disorders in old mice. These included age-related declines in cardiac regenerative capacity, frailty, muscle weakness, reduced daily activity, decreased running endurance on a treadmill, and osteoporosis [3,5,9,28,32]. Additionally, in old mice, D + Q delayed age-related diseases as a group and extended their remaining lifespan by 36% [28]. In mouse models of age- and senescence-related chronic diseases, D + Q alleviated metabolic dysfunction in high fat diet-fed as well as genetically obese mice (in part by decreasing insulin resistance), DKD manifested by podocyte dysfunction and proteinuria, high fat diet-induced renal fibrosis, renal cortical hypo‑oxygenation, and increased creatinine, hepatic steatosis, neuropsychiatric dysfunction in high fat-fed and genetically obese mice, high fat diet-induced cardiac dysfunction and vascular hyporeactivity, Alzheimer's-like dementia in mice with brain tau or β-amyloid protein aggregates, failure of the arteriovenous fistulae used for vascular access during hemodialysis, bleomycin-induced pulmonary fibrosis (a mouse model of IPF), and hyperoxia-induced pulmonary dysfunction, among others [7,9,20,23,24,[32], [33], [34], [35], [36], [37]]. Thus, senolytic agents might have the potential to delay, prevent, or treat age-related diseases as a group, instead of one-at-a-time. To advance toward this goal, it is critical to focus on developing senolytic agents that are safe and effective when administered systemically (as opposed to locally by injection) in humans.

After we reported the first senolytic drugs, D + Q [32], a number of additional senolytics were found by us and others [37,[64], [65], [66], [67], [68], [69], [70]]. For example, based on our report that BCL-xL is an endothelial cell SCAP pathway target in early 2015 [32], we subsequently found that the BCL-2 family inhibitor, Navitoclax (N; also known as ABT-263), and the more specific BCL-xL inhibitors, A1331852 and A1155463, are, like Q, senolytic in the case of senescent human endothelial cells, but not senescent human adipocyte progenitors [64,71]. Thus, the range of senescent cells targeted by BCL-xL inhibitors on their own is restricted. Our rationale for using D + Q as opposed to other drugs in the trial reported here follows. D + Q is more specifically senolytic than other drugs that act as “panolytics”, including some agents that bind MDM2, preventing p53 destabilization (e.g., Nutlin3a) and some BCL-2 family inhibitors (e.g., N). Some such agents, for example nutlins, can cause severe (class 3/4) off-target reductions in non-senescent cell types, including platelet deficiency causing bleeding, neutropenia causing infections, and deficiencies of other cell types [72,73]. This impact on multiple cell types (over and above senescent cells) of drugs that act principally on MDM2 can make it difficult to attribute clinical effects of such drugs to selective targeting of senescent cells (as opposed to non-senescent cells). While D can also sometimes cause transient and reversible platelet deficiency, this is uncommon. Even with hematological malignancies that themselves can cause platelet depletion, significant (class 3/4) bleeding occurs in <1% of patients on continuous D treatment. This usually only occurs after months of uninterrupted D administration, unlike the intermittent D regimen that we found here to be senolytic (Web Reference 1). Additionally, some senolytics (e.g., Q) and panolytic drugs (e.g., N) do not target certain types of senescent cells, such as senescent human adipocyte progenitors [71], which are a major senescent cell type in adipose tissue, and which were studied in the clinical trial reported here, another reason for combining D with Q in our study. A benefit of D + Q is that this senolytic combination more comprehensively disables SCAP networks than N, increasing the range of senescent cell types selectively eliminated by D + Q [32]. We therefore studied systemic administration of D + Q as opposed to panolytic drugs (e.g., Nutlin3a), since the latter may have to be injected locally instead of systemically to avoid adverse effects caused by their off-target depletion of non-senescent cell types.

Many more clinical trials for different indications and with different senolytic drugs and combinations need to be completed to confirm the findings from the three clinical trials of senolytics reported in peer-reviewed journals so far. These types of clinical studies include later phase trials, such as the randomized, controlled trial we and collaborators are currently conducting using D + Q for IPF (NCT02874989) or frailty in elderly women using Fisetin (NCT03325322), an earlier phase trial of D + Q for the accelerated ageing-like state following bone marrow transplantation (NCT02652052), completion of our trial with preliminary results reported here of D + Q for renal insufficiency and stem cell dysfunction in advanced diabetes (NCT02848131), trials for the many other age- and cellular senescence-related disorders using different senolytic agents and combinations that we are about to begin, and trials by others that have yet to be reported following peer review, such as one in which an agent that acts on principally MDM2 is injected into the knees of subjects with osteoarthritis (NCT03513016).

By conducting trials across different age- and senescence-related conditions and using different sets of senolytic agents, the Geroscience Hypothesis [21,74,75] can be tested in humans. This hypothesis posits that by targeting a fundamental ageing process such as cellular senescence, multiple age-related disorders can be delayed, prevented, or alleviated as a group, instead of one-at-a-time. This novel approach will be much more like that involved in developing an antibiotic than the usual one-drug, one-target, one-disease approach that has dominated the biomedical drug development field for the past few decades. The goal of senolytics is by definition to target senescent cells. It is not to identify a single molecular target and to develop a drug that affects that single molecular target. This could cause apoptosis in multiple cell types, including non-senescent cells, or restrict elimination to a small subset of senescent cells that express that particular molecular target. In the current clinical trial we show that D + Q is effective in decreasing senescent cells, the first direct evidence that senolytics are effective in humans.

Although we are optimistic about the prospects for introducing senolytics and other agents that modulate fundamental ageing processes into clinical practice in the future, particularly in the near future for serious diseases for which there are currently no effective interventions, we must conclude with a note of caution. The field of senolytics is new. The first clinical trial of senolytic agents was only reported in January 2019. The findings reported here are preliminary results from an ongoing clinical trial of senolytics for treating dysfunction in patients with diabetic chronic kidney disease. Fewer than 150 subjects have been treated with these drugs in the context of clinical trials that we are aware of so far. In addition to side effects related to individual senolytic drugs known from other contexts in which those drugs have been used, there could turn out to be serious side-effects of senolytics as a class, which are not yet known. We caution against the use of senolytic agents outside the context of clinical trials until more is known about their effects and side effects.

## Funding sources

This work was supported by NIH grants DK109134 (L.J.H.), DK118120 (S.M.H.), DK120292 (L.O.L.), AG013925 (J.L.K.), AG062413 (Project 1; J.L.K.; Project 2: S.K.; Project 3: N.K.L.), the Translational Geroscience Network (AG061456: J.L.K.), DK45343 (M.D.J.), DK40484 (M.D.J.), and TR002377 (S.K.; Mayo Clinic Center for Translational Science Activities), Robert and Arlene Kogod, Satellite Healthcare (L.J.H.), the Connor Group (J.L.K.), Robert J. and Theresa W. Ryan (J.L.K.), and the Glenn (N.K.L.), Ted Nash Long Life (J.L.K.), and Noaber Foundations (J.L.K.). The study sponsors had no role in in the collection, analysis, or interpretation of data; in writing the report; or in the decision to submit the paper for publication. T.T. and J.L.K. confirm they had full access to all the data in the study and had final responsibility for the decision to submit for publication.

## Author contributions

Study concept, design, and overall supervision: JLK, TT, LJH.

Prepared manuscript: JLK, LJH, TT, LGPLP.

Edited manuscript: JLK, LJH, EOWG, TT, LGPLP, SK, RJP, MDJ, SKH, SAB, TKE, SMH, DMK, NKL, KMM, SCT, QJ, YZ, JFP.

Obtained funding: JLK, LJH.

Adipose tissue and skin biopsies: TJM, TAK.

Regulatory approvals and oversight: LJH, TKE, KMM, SH, JLK.

Patient enrollment, consenting, and clinical assessments: LJH, DKL, SMH, SCT, SAB, EOWG, TLV, JLK.

Conducted adipocyte progenitor replication assays and harvesting: KLJ, IMS.

Conducted immunohistochemical, SAβgal, and blood laboratory analyses: TT, LGPLP, AX, QJ, MDJ, SGV, ABL, JFP, DMK, KKS.

Data analysis: JLK, TT, LJH, LGPLP, RJP.

## Declaration of Competing Interest

J.L.K., T.T., Y.Z., and N.K.L. have a financial interest related to this research. Patents on senolytic drugs are held by Mayo Clinic. This research has been reviewed by the Mayo Clinic Conflict of Interest Review Board and was conducted in compliance with Mayo Clinic Conflict of Interest policies. No conflicts of interest, financial or otherwise, are declared by the other authors.

## References

[1] Hayflick L., Moorehead P. (1961). The serial cultivation of human diploid strains. Exp Cell Res.

[2] Kirkland J.L., Tchkonia T. (2017). Cellular senescence: a translational perspective. EBioMedicine..

[3] Lewis-McDougall F.C., Ruchaya P.J., Domenjo-Vila E., Shin Teoh T., Prata L., Cottle B.J. (2019). Aged-senescent cells contribute to impaired heart regeneration. Aging Cell.

[4] Kirkland J.L., Hollenberg C.H., Gillon W.S. (1990). Age, anatomic site, and the replication and differentiation of adipocyte precursors. Am J Physiol.

[5] Farr J.N., Xu M., Weivoda M.M., Monroe D.G., Fraser D.G., Onken J.L. (2017). Targeting cellular senescence prevents age-related bone loss in mice. Nat Med.

[6] Justice J.N., Gregory H., Tchkonia T., LeBrasseur N.K., Kirkland J.L., Kritchevsky S.B. (2018). Cellular senescence biomarker p16^INK4a+^ cell burden in thigh adipose is associated with poor physical function in older women. T J Gerontol A Biol Sci Med Sci.

[7] Ogrodnik M., Miwa S., Tchkonia T., Tiniakos D., Wilson C.L., Lahat A. (2017 Jun 13). Cellular senescence drives age-dependent hepatic steatosis. Nat Commun.

[8] Farr J.N., Fraser D.G., Wang H., Jaehn K., Ogrodnik M.B., Weivoda M.M. (2016). Identification of senescent cells in the bone microenvironment. J Bone Miner Res.

[9] Roos C.M., Zhang B., Palmer A.K., Ogrodnik M.B., Pirtskhalava T., Thalji N.M. (2016). Chronic senolytic treatment alleviates established vasomotor dysfunction in aged or atherosclerotic mice. Aging Cell.

[10] Xu M., Palmer A.K., Ding H., Weivoda M.M., Pirtskhalava T., White T.A. (2015). Targeting senescent cells enhances adipogenesis and metabolic function in old age. eLife..

[11] Xu M., Tchkonia T., Ding H., Ogrodnik M., Lubbers E.R., Pirtskhalava T. (2015). JAK inhibition alleviates the cellular senescence-associated secretory phenotype and frailty in old age. P Proc Natl Acad Sci (USA).

[12] Zhu Y., Armstrong J.L., Tchkonia T., Kirkland J.L. (2014). Cellular senescence and the senescent secretory phenotype in age-related chronic diseases. Curr Opin Clin Nutr Metab Care.

[13] Tchkonia T., Morbeck D.E., von Zglinicki T., van Deursen J., Lustgarten J., Scrable H. (2010). Fat tissue, aging, and cellular senescence. Aging Cell.

[14] Kirkland J.L., Dobson D.E. (1997). Preadipocyte function and aging: links between age-related changes in cell dynamics and altered fat cell function. J Amer Geriatr Soc.

[15] Tchkonia T., Giorgadze N., Pirtskhalava T., Thomou T., Villaret A., Bouloumie A. (2009). Cellular senescence and inflammation in obesity. Obesity.

[16] Villaret A., Galitzky J., Decaunes P., Esteve D., Marques M.A., Sengenes C. (2010). Adipose tissue endothelial cells from obese human subjects: differences among depots in angiogenic, metabolic, and inflammatory gene expression and cellular senescence. Diabetes..

[17] Minamino T., Orimo M., Shimizu I., Kunieda T., Yokoyama M., Ito T. (2009). A crucial role for adipose tissue p53 in the regulation of insulin resistance. N Nat Med.

[18] Docherty M.H., O'Sullivan E.D., Bonventre J.V., Ferenbach D.A. (2019). Cellular senescence in the kidney. J Am Soc Nephrol.

[19] Dai L., Qureshi A.R., Witasp A., Lindholm B., Stenvinkel P. (2019). Early vascular ageing and cellular senescence in chronic kidney disease. Comput Struct Biotechnol J.

[20] Kim S.R., Jiang K., Ogrodnik M., Chen X., Zhu X.Y., Lohmeier H. (2019). Increased renal cellular senescence in murine high-fat diet: effect of the senolytic drug quercetin. Transl Res.

[21] Kirkland J.L., Tchkonia T., Zhu Y., Niedernhofer L.J., Robbins P.D. (2017 Oct). The clinical potential of senolytic drugs. J Amer Geriatr Soc.

[22] Tchkonia T., Kirkland J.L. (2018). Aging, cell senescence, and chronic disease: emerging therapeutic strategies. JAMA.

[23] Palmer A.K., Xu M., Zhu Y., Pirtskhalava T., Weivoda M.M., Hachfeld C.M. (2019). Targeting senescent cells alleviates obesity-induced metabolic dysfunction. Aging Cell.

[24] Ogrodnik M., Zhu Y., Langhi L.G.P., Tchkonia T., Kruger P., Fielder E. (2019). Obesity-induced cellular senescence drives anxiety and impairs neurogenesis. Cell Metab.

[25] Musi N., Valentine J.M., Sickora K.R., Baeuerle E., Thompson C.S., Shen Q. (2018). Tau protein aggregation is associated with cellular senescence in the brain. Aging Cell.

[26] Coppé J.P., Patil C., Rodier F., Sun Y., Muñoz D.P., Goldstein J. (2008). Senescence-associated secretory phenotypes reveal cell-nonautonomous functions of oncogenic RAS and the p53 tumor suppressor. PLoS Biol.

[27] Xu M., Bradley E.W., Weivoda M.M., Hwang S.M., Pirtskhalava T., Decklever T. (2017). Transplanted senescent cells induce an osteoarthritis-like condition in mice. J Gerontol A Biol Sci Med Sci.

[28] Xu M., Pirtskhalava T., Farr J.N., Weigand B.M., Palmer A.K., Weivoda M.M. (2018). Senolytics improve physical function and increase lifespan in old age. Nat Med.

[29] Wang E. (1995). Senescent human fibroblasts resist programmed cell death, and failure to suppress BCL2 is involved. Cancer Res.

[30] Kirkland J.L., Tchkonia T. (2015). Clinical strategies and animal models for developing senolytic agents. Exp Gerontol.

[31] Krishnamurthy J., Torrice C., Ramsey M.R., Kovalev G.I., Al-Regaiey K., Su L. (2004). Ink4a/Arf expression is a biomarker of aging. J Clin Invest.

[32] Zhu Y., Tchkonia T., Pirtskhalava T., Gower A., Ding H., Giorgadze N. (2015). The Achilles' heel of senescent cells: from transcriptome to senolytic drugs. Aging Cell.

[33] Schafer M.J., White T.A., Iijima K., Haak A.J., Ligresti G., Atkinson E.J. (2017). Cellular senescence mediates fibrotic pulmonary disease. Nat Commun.

[34] Parikh P., Britt R.D., Manlove L.J., Wicher S.A., Roesler A., Ravix J. (2019). Hyperoxia-induced cellular senescence in fetal airway smooth muscle cells. Am J Respir Cell Mol Biol.

[35] Nath K.A., O'Brien D.R., Croatt A.J., Grande J.P., Ackerman A.W., Nath M.C. (2018). The murine dialysis fistula model exhibits a senescence phenotype: pathobiologic mechanisms and therapeutic potential. Am J Physiol Renal Physiol.

[36] Zhang P., Kishimoto Y., Grammatikakis I., Gottimukkala K., Cutler R.G., Zhang S. (2019). Senolytic therapy alleviates Abeta-associated oligodendrocyte progenitor cell senescence and cognitive deficits in an Alzheimer's disease model. Nat Neurosci.

[37] Fuhrmann-Stroissnigg H., Ling Y.Y., Zhao J., McGowan S.J., Stripay J.L., Gregg S. (2017). Identification of HSP90 inhibitors as senolytics for extending healthspan. Nat Commun.

[38] Christopher L.J., Cui D., Wu C., Luo R., Manning J.A., Bonacorsi S.J. (2008). Metabolism and disposition of dasatinib after oral administration to humans. Drug Metab Dispos.

[39] Graefe E.U., Wittig J., Mueller S., Riethling A.K., Uehleke B., Drewelow B. (2001). Pharmacokinetics and bioavailability of quercetin glycosides in humans. J Clin Pharmacol.

[40] Justice J.N., Nambiar A.M., Tchkonia T., LeBrasseur N.K., Pascual R., Hashmi S.K. (2019). Senolytics in idiopathic pulmonary fibrosis: results from a first-in-human, open-label, pilot study. EBioMedicine..

[41] Martyanov V., Whitfield M.L., Varga J. (2019). Senescence signature in skin biopsies from systemic sclerosis patients treated with senolytic therapy: potential predictor of clinical response?. Arthritis Rheumatol.

[42] Verzola D., Gandolfo M.T., Gaetani G., Ferraris A., Mangerini R., Ferrario F. (2008). Accelerated senescence in the kidneys of patients with type 2 diabetic nephropathy. Am J Physiol Renal Physiol.

[43] Liu J., Yang J.R., Chen X.M., Cai G.Y., Lin L.R., He Y.N. (2015). Impact of ER stress-regulated ATF4/p16 signaling on the premature senescence of renal tubular epithelial cells in diabetic nephropathy. Am J Physiol Cell Physiol.

[44] Satriano J., Mansoury H., Deng A., Sharma K., Vallon V., Blantz R.C. (2010). Transition of kidney tubule cells to a senescent phenotype in early experimental diabetes. Am J Physiol Cell Physiol.

[45] Schneider C.A., Rasband W.S., Eliceiri K.W. (2012). NIH image to ImageJ: 25 years of image analysis. Nat Methods.

[46] Schindelin J., Arganda-Carreras I., Frise E., Kaynig V., Longair M., Pietzsch T. (2012). Fiji: an open-source platform for biological-image analysis. Nat Methods.

[47] Morgan-Bathke M., Harteneck D., Jaeger P., Sondergaard E., Karwoski R., Espinosa De Ycaza A. (2017). Comparison of methods for analyzing human adipose tissue macrophage content. Obesity.

[48] Eirin A., Zhu X.Y., Krier J.D., Tang H., Jordan K.L., Grande J.P. (2012). Adipose tissue-derived mesenchymal stem cells improve revascularization outcomes to restore renal function in swine atherosclerotic renal artery stenosis. Stem Cells.

[49] Saad A., Dietz A.B., Herrmann S.M.S., Hickson L.J., Glockner J.F., McKusick M.A. (2017). Autologous mesenchymal stem cells increase cortical perfusion in renovascular disease. J Am Soc Nephrol.

[50] Wang A.S., Dreesen O. (2018). Biomarkers of cellular senescence and skin aging. Front Genet.

[51] Bernardes de Jesus B., Blasco M.A. (2012). Assessing cell and organ senescence biomarkers. Circ Res.

[52] Murano I., Barbatelli G., Parisani V., Latini C., Muzzonigro G., Castellucci M. (2008). Dead adipocytes, detected as crown-like structures, are prevalent in visceral fat depots of genetically obese mice. J Lipid Res.

[53] Mitsui Y., Schneider E.L. (1976). Relationship between cell replication and volume in senescent human diploid fibroblasts. Mech Ageing Dev.

[54] Schneider E.L. (1979). Cell replication and aging: *in vitro* and *in vivo* studies. Fed Proc.

[55] Zhu Y., Tchkonia T., Stout M.B., Giorgadze N., Wang L., Li P.W. (2015). Inflammation and the depot-specific secretome of human preadipocytes. Obesity..

[56] Tchkonia T., Cartwright M., Wise B., Pirtskhalava T., Karagiannides I., Shpilman A. (2007). Increased TNFa and CCAAT/enhancer binding protein homologous protein (CHOP) with aging predispose preadipocytes to resist adipogenesis. Am J Physiol.

[57] Tchkonia T., Lenburg M., Thomou T., Giorgadze N., Frampton G., Pirtskhalava T. (2007). Identification of depot-specific human fat cell progenitors through distinct expression profiles and developmental gene patterns. Am J Physiol.

[58] Doebel T., Voisin B., Nagao K. (2017). Langerhans cells - the macrophage in dendritic cell clothing. Trends Immunol.

[59] Hall B.M., Balan V., Gleiberman A.S., Strom E., Krasnov P., Virtuoso L.P. (2017). p16(Ink4a) and senescence-associated beta-galactosidase can be induced in macrophages as part of a reversible response to physiological stimuli. Aging (Albany NY).

[60] Waaijer M.E., Parish W.E., Strongitharm B.H., van Heemst D., Slagboom P.E., de Craen A.J. (2012). The number of p16^INK4a^ positive cells in human skin reflects biological age. Aging Cell.

[61] Bhalla S., Tremblay D., Mascarenhas J. (2016). Discontinuing tyrosine kinase inhibitor therapy in chronic myelogenous leukemia: current understanding and future directions. Clin Lymphoma Myeloma Leuk.

[62] Fox L.C., Cummins K.D., Costello B., Yeung D., Cleary R., Forsyth C. (2017). The incidence and natural history of dasatinib complications in the treatment of chronic myeloid leukemia. Blood Adv.

[63] La Rosee P., Martiat P., Leitner A., Klag T., Muller M.C., Erben P. (2013). Improved tolerability by a modified intermittent treatment schedule of dasatinib for patients with chronic myeloid leukemia resistant or intolerant to imatinib. Ann Hematol.

[64] Zhu Y., Doornebal E.J., Pirtskhalava T., Giorgadze N., Wentworth M., Fuhrmann-Stroissnigg H. (2017). New agents that target senescent cells: the flavone, fisetin, and the BCL-XL inhibitors, A1331852 and A1155463. Aging (Albany NY).

[65] Yousefzadeh M.J., Zhu Y., McGowan S.J., Angelini L., Fuhrmann-Stroissnigg H., Xu M. (2018 Oct). Fisetin is a senotherapeutic that extends health and lifespan. EBioMedicine..

[66] Wang Y., Chang J., Liu X., Zhang X., Zhang S., Zhou D. (2016). Discovery of piperlongumine as a potential novel lead for the development of senolytic agents. Aging (Milano).

[67] Ozsvari B., Nuttall J.R., Sotgia F., Lisanti M.P. (2018). Azithromycin and Roxithromycin define a new family of "senolytic" drugs that target senescent human fibroblasts. Aging (Milano).

[68] Cherif H., Bisson D.G., Jarzem P., Weber M., Ouellet J.A., Haglund L. (2019). Curcumin and o-vanillin exhibit evidence of senolytic activity in human IVD cells in vitro. J Clin Med.

[69] Li W., He Y., Zhang R., Zheng G., Zhou D. (2019). The curcumin analog EF24 is a novel senolytic agent. Aging (Milano).

[70] Samaraweera L., Adomako A., Rodriguez-Gabin A., McDaid H.M. (2017). A novel indication for panobinostat as a senolytic drug in NSCLC and HNSCC. Sci Rep.

[71] Zhu Y., Tchkonia T., Fuhrmann-Stroissnigg H., Dai H.M., Ling Y.Y., Stout M.B. (2015). Identification of a novel senolytic agent, navitoclax, targeting the Bcl-2 family of anti-apoptotic factors. Aging Cell.

[72] Kaefer A., Yang J., Noertersheuser P., Mensing S., Humerickhouse R., Awni W. (2014). Mechanism-based pharmacokinetic/pharmacodynamic meta-analysis of navitoclax (ABT-263) induced thrombocytopenia. Cancer Chemother Pharmacol.

[73] Mahfoudhi E., Lordier L., Marty C., Pan J., Roy A., Roy L. (2016). P53 activation inhibits all types of hematopoietic progenitors and all stages of megakaryopoiesis. Oncotarget..

[74] Justice J., Miller J.D., Newman J.C., Hashmi S.K., Halter J., Austad S.N. (2016). Frameworks for proof-of-concept clinical trials of interventions that target fundamental aging processes. J Gerontol A Biol Sci Med Sci.

[75] Kennedy B.K., Berger S.L., Brunet A., Campisi J., Cuervo A.M., Epel E.S. (2014). Geroscience: linking aging to chronic disease. Cell..

[journal] http://packageinserts.bms.com/pi/pi_sprycel.pdf,

