# Corrigendum to ‘Senolytics decrease senescent cells in humans: Preliminary report from a clinical trial of Dasatinib plus Quercetin in individuals with diabetic kidney disease’ EBioMedicine 47 (2019) 446–456

**DOI:** 10.1016/j.ebiom.2019.12.004

**Published:** 2020-01-23

**Authors:** LaTonya J. Hickson, Larissa G.P. Langhi Prata, Shane A. Bobart, Tamara K. Evans, Nino Giorgadze, Shahrukh K. Hashmi, Sandra M. Herrmann, Michael D. Jensen, Qingyi Jia, Kyra L. Jordan, Todd A. Kellogg, Sundeep Khosla, Daniel M. Koerber, Anthony B. Lagnado, Donna K. Lawson, Nathan K. LeBrasseur, Lilach O. Lerman, Kathleen M. McDonald, Travis J. McKenzie, João F. Passos, Robert J. Pignolo, Tamar Pirtskhalava, Ishran M. Saadiq, Kalli K. Schaefer, Stephen C. Textor, Stella G. Victorelli, Tammie L. Volkman, Ailing Xue, Mark A. Wentworth, Erin O. Wissler Gerdes, David B. Allison, Stephanie L. Dickinson, Keisuke Ejima, Elizabeth J. Atkinson, Marc Lenburg, Yi Zhu, Tamara Tchkonia, James L. Kirkland

**Affiliations:** a Cellular Senescence and Translation and Pharmacology Programs, Robert and Arlene Kogod Center on Aging, Mayo Clinic, Rochester, MN, United States; b Division of Geriatric Medicine and Gerontology, Department of Medicine, Mayo Clinic, Rochester, MN, United States; c Division of Nephrology and Hypertension, Department of Medicine, Mayo Clinic, Rochester, MN, United States; d Department of Medicine Clinical Trials Unit, Department of Medicine, Mayo Clinic, Rochester, MN, United States; e Division of Hematology, Department of Medicine, Mayo Clinic, Rochester, MN, United States; f Division of Endocrinology, Department of Medicine, Mayo Clinic, Rochester, MN, United States; g Department of Surgery, Mayo Clinic, Rochester, MN, United States; h Department of Physiology and Biomedical Engineering, Mayo Clinic, Rochester, MN, United States; i Division of Hospital Medicine, Department of Medicine, Mayo Clinic, Rochester, MN, United States; j Department of Physical Medicine and Rehabilitation, Mayo Clinic, Rochester, MN, United States; k Office of Research Regulatory Support, Mayo Clinic, Rochester, MN, United States; l Department of Epidemiology and Biostatistics, School of Public Health, Indiana University – Bloomington, Bloomington, IN, United States; m Division of Biomedical Statistics and Informatics, Department of Health Sciences Research, Mayo Clinic, Rochester, MN, United States; n Section of Computational Biomedicine, Boston University School of Medicine, Boston, MA, United States; o Division of General Internal Medicine, Department of Medicine, Mayo Clinic, Rochester, MN, United States

In this article, a re-analysis of the raw data has been conducted, which means that some conclusions presented in the paper have changed. The authors have written a Corrigendum which is appended (Appendix 1).

In the interests of transparency, they have included de-identified data from the original article in Appendix 2.

## Appendix Supplementary materials

Image, application 1

Image, application 1

