# Supplementary material for: Corrigendum to ‘Senolytics decrease senescent cells in humans: Preliminary report from a clinical trial of Dasatinib plus Quercetin in individuals with diabetic kidney disease’ EBioMedicine 47 (2019) 446–456
Source: eBioMedicine. 2020 Jan 23;52:102595. doi: 10.1016/j.ebiom.2019.12.004 (PMC6994619; doi:10.1016/j.ebiom.2019.12.004)
Supplement: Supplementary file 1 [file mmc1.docx]

Senolytics Decrease Senescent Cells in Humans: Preliminary Report from a Clinical Trial of Dasatinib plus Quercetin in Individuals with Diabetic Kidney Disease, EBioMedicine. 2019 Sep;47:446-456. doi: 10.1016/j.ebiom.2019.08.069. *Corrigendum*

**Introduction**

The central hypothesis tested in our article is that a brief course of the senolytic drug combination, Dasatinib plus Quercetin (D+Q), can reduce senescent cell abundance in humans, specifically focusing on targeting adipose tissue in subjects with diabetes and kidney dysfunction, a condition in which adipose tissue senescent cell burden is known to be increased (ref 1). We speculated that any decline in senescent cell abundance should remain evident for at least 10 days after completion of senolytic treatment. This was based on the points that the elimination half-life of D+Q is brief, resulting in removal within two days, and that senescent cells take two or more weeks to develop, at least in culture.

In 11 subjects with diabetes and kidney dysfunction, we focused on determining whether adipose tissue senescent cells are decreased 11 days after completing a 3 day course of D+Q (*i.e*. by Day 14). We had adipose tissue for immunohistochemistry from 9 of these 11 subjects. We reported that senescent cells were decreased in the Day 14 adipose tissue biopsies, together with decreases in the macrophages (CD68^+^ cells) and crown-like structures (CLS) to which senescent cells are linked. Besides the decline in senescent cells in adipose tissue, the tissue central to our hypothesis, we noted changes in ancillary measures, including declines in circulating factors that have been linked to senescence.

In the article, we showed data after transforming the Day 0 and Day 14 values within subjects as %. Based on the advice of colleagues and readers, we show data and statistics without that transformation and show the raw data in the following Figures and Appendix. We present analyses of these data conducted and/or reviewed by professional statisticians (see acknowledgements). Also, in the article, we used unpaired T-tests (instead of pre-planned paired T-tests), since unpaired T-tests can be more conservative than paired T-tests. Here, we show data analyzed using paired T-tests and statistical analyses of composite scores.

Here, re-analysis of the raw data shows that the key marker of senescence, p16^INK4A+^ cells, is significantly decreased in adipose tissue 11 days after completing a 3 day course of D+Q compared to before treatment using paired T-tests (p=0.017; Fig. 1; see Adipose Tissue Results Raw Data below). Also consistent with effects of senolytics on adipose tissue in mice, in these subjects CD68^+^ cells (macrophages) and CLS were significantly decreased at day 14 in analyses of the raw data (p=0.007 and p=0.004, respectively; Fig. 2; see Adipose Tissue Results Raw Data below). Since determining if D+Q decreases senescent cell abundance in adipose tissue of humans (diabetic subjects) is the primary intent of our preliminary report, a joint analysis of 6 adipose tissue cellular senescence-associated measures was done for the 9 subjects in the original article from whom immunohistochemical data were available (p16^INK4A+^ cells, SA-βgal^+^ cells, p21^CIP1+^ cells, CD68^+^ cells, CLS, preadipocyte proliferation). This harmonic mean p-value (HMP) showed a statistically significant effect of D+Q (p=0.020; Fig. 3; see Grouped Analysis below).

When data from the 9 out of the 11 subjects in the original article from whom adipose tissue immunohistochemical data were also available were analyzed without transformation, skin senescence markers were not statistically decreased (Fig. 4; raw data are in the attached data file).

We tested 10 blood senescence-associated secretory phenotype (SASP) factors 11 days after completing 3 days of D+Q in the original article. In the 9 subjects for whom these 10 SASP factors were reported, the brief exposure to D+Q significantly attenuated the blood SASP using a composite score analysis (p=0.003). Blood levels of MMP-12 were reduced significantly by D+Q (p=0.034; N=8; log-transformed, paired T-test; Fig. 5; see SASP Factors below). SASP factor raw data are in the attached data file.

**Revised Tables and Figures**

**Table 1.** Baseline demographic and clinical variables of diabetic kidney disease participants treated with a single 3-day oral course of D+Q.

**Variable** **Participants Treated with D+Q (N=11^a^)**

Age, years 69.3 (2.6)

Sex 9 male, 2 female

Race 10 white, 1 Hispanic

BMI, kg/m^2^ 33.2 (1.9)

eGFR, mL/min/1·73m^2^ 28.2 (2.1)

Diabetes Insulin therapy alone 5/11

Oral glucose lowering therapy alone 4/11

Both insulin and oral glucose lowering therapy 2/11

Data represent Mean (SEM).

BMI: body mass index; eGFR: estimated glomerular filtration rate.

^a^ Corrected footnote for the legend: adipose tissue samples for immunohistochemical analysis from 2 of the 11 subjects in the study (subjects 10 and 11: ages 70 & 75 years, BMI 31.1 & 29.4 kg/m^2^, eGFR 38 & 29 mL/min/1.73m^2^, males, white, diabetic on glucose lowering therapy alone) were damaged during preparation, so immunohistochemistry was not done, but it was possible to conduct analyses for adipocyte progenitor proliferation (shown in Figure 3 of the original article), SA β-gal, and epidermal markers (not shown in the original article).


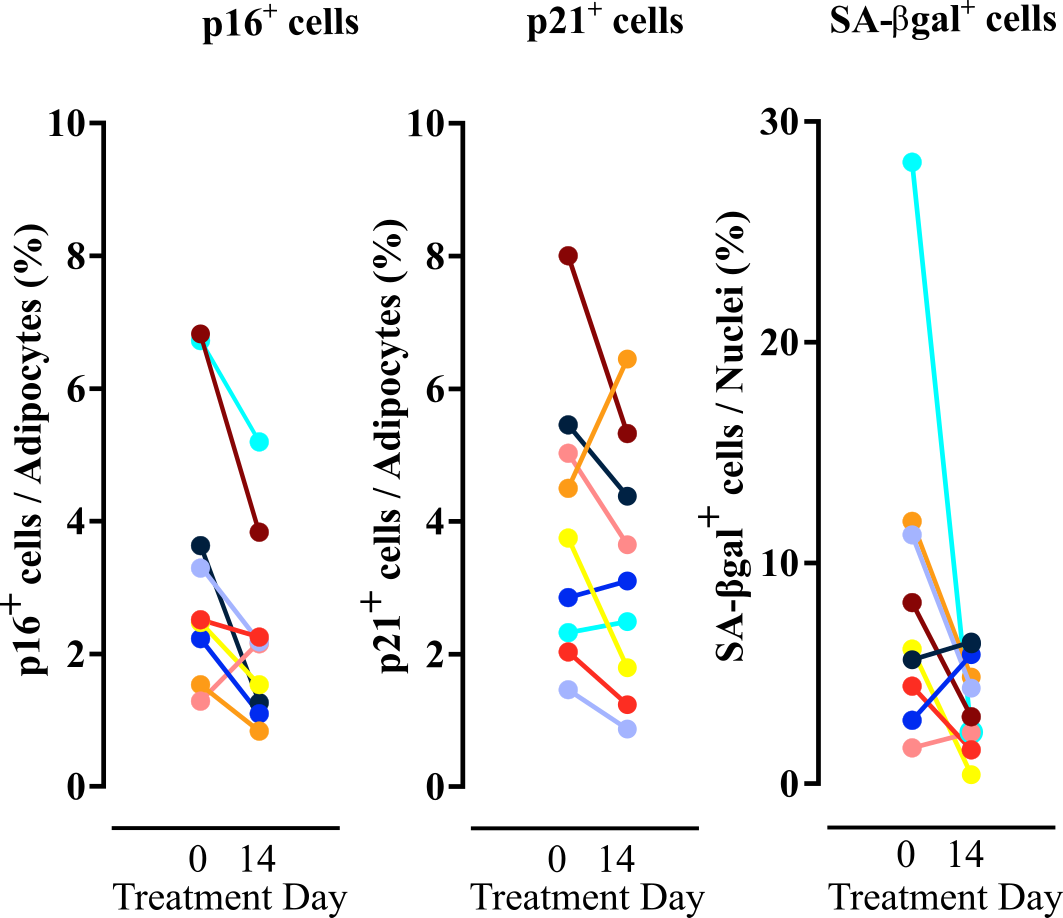


**Figure 1.** D+Q effects on human adipose tissue senescent cells. Left panel: D+Q significantly reduced (p=0.017; N=9 subjects; paired T-test) abdominal subcutaneous adipose tissue p16^INK4A+^ cells in adipose tissue sections at Day 14 (11 days after the last dose of a 3-day course of the senolytics) *vs.* baseline (Day 0). At Day 0, there were on average 3.18±0.69 p16^INK4A+^ cells/100 adipocytes (means of 30 fields [400×300μm] at 40× magnification). Middle panel: p21^CIP1+^ cells. At baseline (Day 0), there were 3.85±0.69 p21^CIP1+^ cells/100 adipocytes (N=9; before *vs*. after D+Q p=0.141, paired 2-tailed T test for log-transformed data). Right panel: there were on average 8.76±2.67 adipose tissue SA-βgal^+^ cells/100 nuclei. If only the 9 subjects in the original article (out of the 11 subjects enrolled and analyzed) are considered, p=0.059, paired T-test before *vs*. after D+Q; see statistical notes below. Colours indicate each individual's values on Days 0 and 14. The same colour is used for each subject across the figures. D+Q had a significant effect in a grouped analysis of these measures (p=0.036; p16^INK4A+^ cells, SA-βgal^+^ cells, and p21^CIP1+^ cells; harmonic mean p-value; see statistical notes below). Raw data for all Figures are in an appended file.


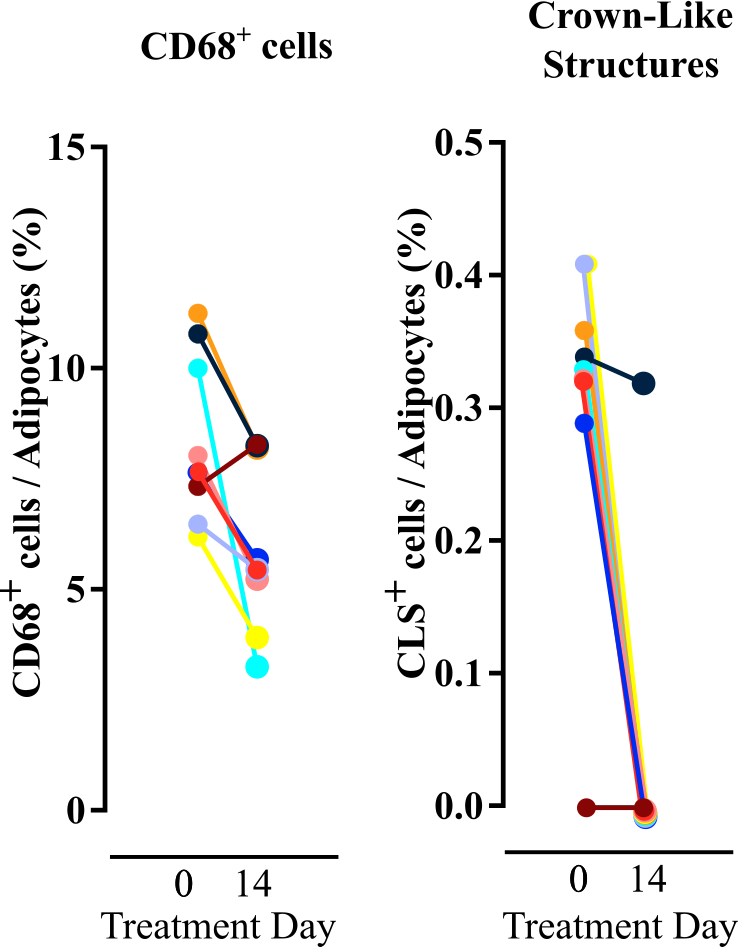


**Figure 2.** D+Q decreases human adipose tissue macrophages and crown-like structures. Left panel: D+Q significantly reduced adipose tissue CD68^+^ macrophages relative to adipocytes (p=0.007; N=9 subjects; paired T-test). D+Q do not directly target human macrophages, but rather the senescent cells that attract them (ref. 2). At baseline, there were on average 8.40±0.62 CD68^+^ macrophages/100 adipocytes (N=9 subjects). Right panel: D+Q reduced adipose tissue crown-like structures (CLS). CLS were significantly decreased by 11 days after D+Q treatment (p=0.004; N=9 subjects; paired T-test for log-transformed data). At baseline (Day 0), there were on average 0.27±0.05 CLS/100 adipocytes (N=9 subjects). Colours indicate each individual's values on Days 0 and 14. See statistical notes below.


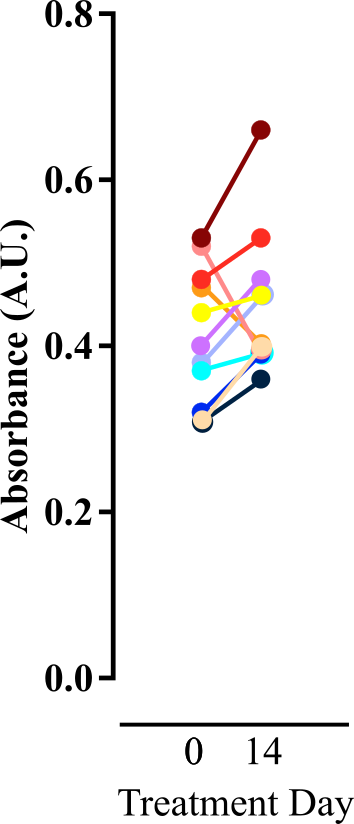


**Figure 3.** Changes in adipocyte progenitor cell density over time following administration of D+Q. Cell density/ time was assayed by tetrazolium uptake in adipocyte progenitors isolated from adipose biopsies acquired before (Day 0) and 14 days after the first dose of the 3-day course of D+Q (Day 14) and cultured in parallel for 3 passages. At baseline (Day 0), cell density/ time was on average 0.41±0.02. Cell density over time is shown for adipocyte progenitors isolated after senolytic treatment compared with adipocyte progenitors isolated before treatment (N=11 subjects; p=0.149; paired T-test). D+Q did have a significant effect in a grouped analysis of p16^INK4A+^ cells, SA-βgal^+^ cells, p21^CIP1+^ cells CD68^+^ cells, crown-like structures, plus adipocyte progenitor proliferation in the 9 subjects reported in the original article (p=0.020; harmonic mean p-value; see statistical notes below). Colours indicate each individual's values on Days 0 and 14.

**Figure 4.** Effect of D+Q on human epidermal senescent cells. Left panel: At baseline (Day 0), there were on average 1.95±0.68 human epidermal basal layer p16^INK4A+^ cells/mm of epidermis (N=9 subjects). Middle panel: At baseline (Day 0), there were on average 1.71±0.34 human epidermal basal layer p21^CIP1+^ cells/mm of epidermis (N=9 subjects). Right panel: D+Q did not substantially change (p=0.545; N=9 subjects; paired T-test for log-transformed data) antigen-presenting CD1a^+^ epidermal Langerhans immune cells. At baseline (Day 0), there were on average 14.55±2.30 CD1a^+^ cells/mm of epidermis (N=9 subjects). Colours indicate each individual's values on Days 0 and 14. See statistical notes below.


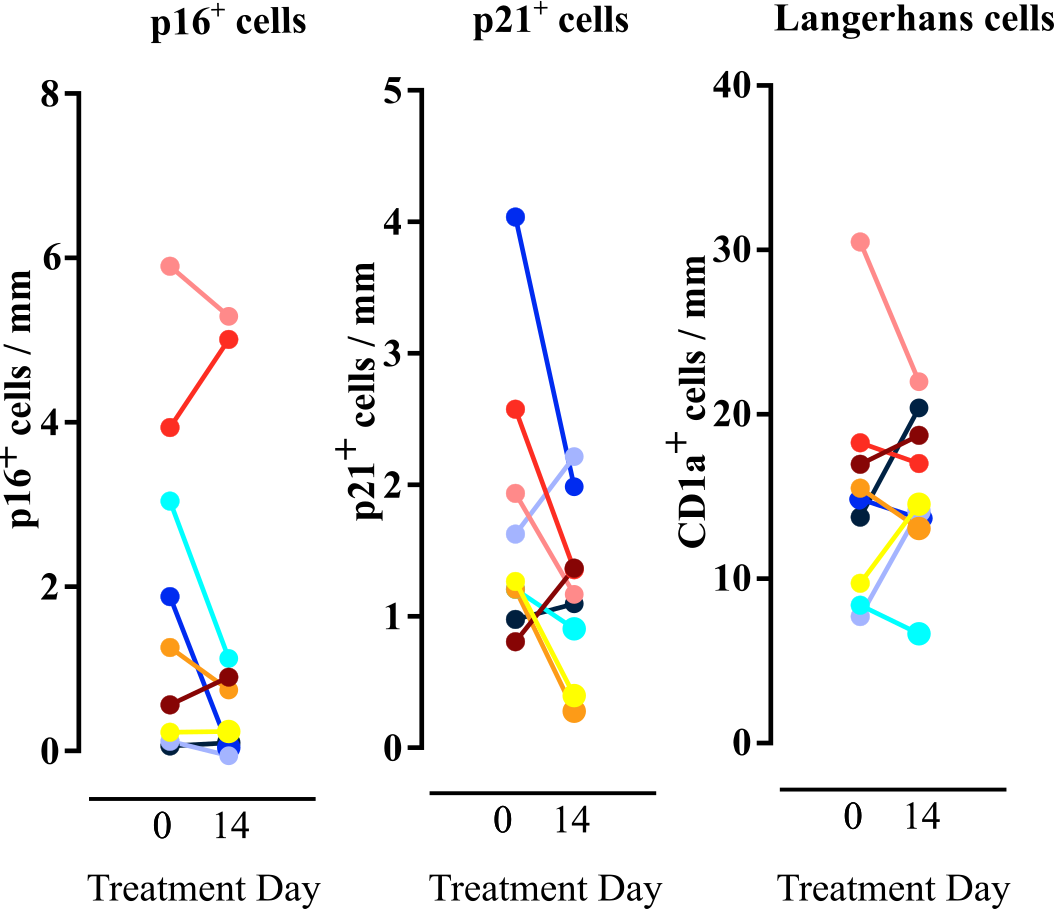


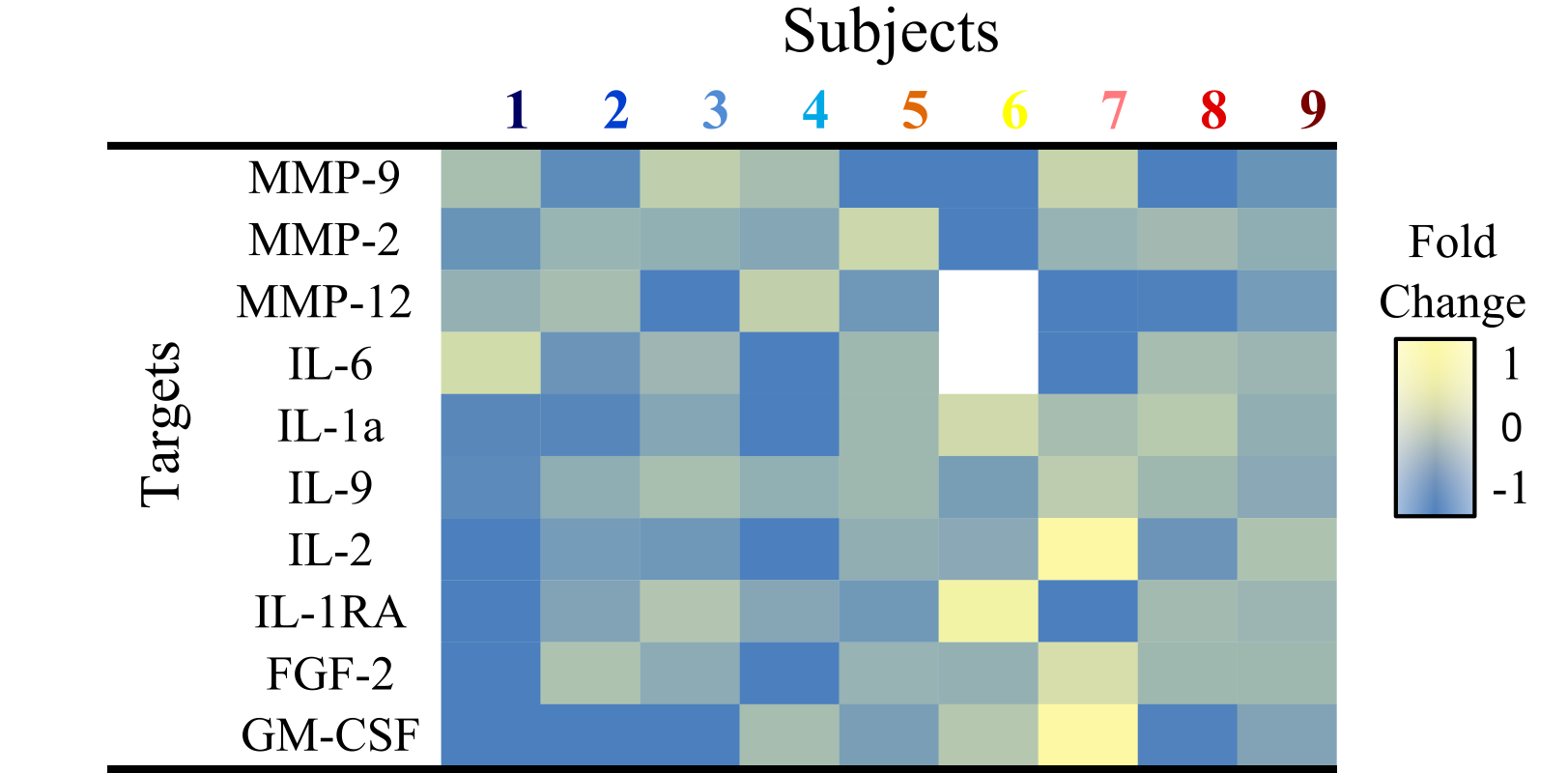


**Figure 5.** D+Q causes a statistically significant overall reduction in plasma SASP factors (p=0.003; N=9; composite score ‒ see statistical notes below). Plasma SASP factors were assayed at baseline (Day 0) and after treatment (Day 14). Colours indicate changes for each individual between Days 0 and 14 (post-treatment/ baseline value; N=9), with blue indicating a decrease, yellow an increase, and white missing values. Values were expressed as log_2_ first so that a value of 0 indicates the SASP factor is unchanged, +1 indicates a 2-fold increase, and -1 indicates a 2-fold decrease. MMP-12 was significantly decreased (N=9; p=0.034; paired T-test for log-transformed data).

**Supplemental Table 1.** Effect of repeating biopsy on adipose tissue SA-βgal^+^ cells.

| Subject | SA-βgal^+^ cells/100 nuclei Day 1 | SA-βgal^+^ cells/100 nuclei Day 15 |
| --- | --- | --- |
| 1a | 5.6 | 4.3 |
| 2a | 1.2 | 1.7 |
| 3a | 3.6 | 4.9 |
| 4a | 2.5 | 1.4 |
| 5a | 4.9 | 3.7 |
| 6a | 9.4 | 12.3 |
| 7a | 2.9 | 3.2 |
| 8a | 1.1 | 1.1 |
| 9a | 2.6 | 2.3 |
| 10a | 0.7 | 1.1 |
| 11a | 5.6 | 8.7 |
| 12a | 3.1 | 4.6 |
| 13a | 0.9 | 2.6 |
| 14a | 6.4 | 6.8 |
| 15a | 2.9 | 1.5 |
| Mean | **3.56** | **4.01** |
| Standard Error | **0.63** | **0.82** |

At completion, the open-label, non-placebo controlled parent study that our preliminary report was based on will have enrolled 30 subjects, 20 given D+Q and 10 no drugs. We did not yet analyze the latter group since at the time of the preliminary report, only a small number of subjects had been enrolled in the non-drug treated group. In the absence of such data, to test whether conducting a second biopsy may cause an artefactual decrease in senescence markers, we analyzed SA-βgal^+^ cells in adipose tissue from 15 subjects with diabetes who were not given senolytics and who were biopsied twice 15 days apart in a different study. In this control experiment, there was no apparent trend to a decrease in SA-βgal^+^ cells in the repeat biopsies.

**Statistical Notes**

All statistical tests are on raw or log-transformed data (not % transformed data) from individual assays. Statistically significant outcomes at p<0.05 are in bold type. All statistical tests and p-values are 2-tailed. Means ± standard error (SE) are reported. Data for studies are available from all 11 subjects except for adipose tissue immunohistochemistry (adipose tissue samples for immunohistochemistry from those 2 subjects were inadequate), but only results reported in the original article are shown below. First, to test the normality of the distribution (which is a necessary assumption for the T-test), Shapiro-Wilk (SW) tests were performed on the difference of the measurements before and after the treatment, as well as skewness and kurtosis statistics (where normally-distributed data have skewness close to 0 and kurtosis close to 1). If the hypothesis of normality was not rejected from SW, we subsequently performed paired T-tests comparing the means before and after treatment. If the hypothesis of normality was rejected, natural log-transformed data were analyzed. As distributions of blood inflammation markers were skewed, the data were log-transformed (natural log) and analyzed. As distributions of SA-βgal cells and CLS for adipose tissue were skewed, the data were log-transformed (natural log) and the same statistical approaches were followed as above.

**Adipose Tissue Results**

**Raw Data**

| Adipose parameter | N | Mean difference (SE) | Shapiro–Wilk p | Paired T-test |  |  |
| --- | --- | --- | --- | --- | --- | --- |
| p16^INK4A+^ cells | | 9^1^ | -1.13 (0.38) | 0.913 | **0.017** | |
| p21^CIP1+^ cells | | 9 | -0.71 (0.43) | 0.973 | 0.141 | |
| SA-βgal | | 9^2^ | -5.46 (2.82) | 0.015 | See below | |
| CD68^+^ cells | | 9 | -2.41 (0.68) | 0.161 | **0.007** |  |
| Proliferation | | 11 | 0.04 (0.02) | 0.088 | 0.149 |  |

**Log Transformed Data**

|  | N | Paired T-test |
| --- | --- | --- |
| SA-βgal | 9^2^ | 0.059 |
| CLS^3^ | 9 | **0.004** |

1. Immunohistochemical data were only available from 9 of the 11 subjects in the original report.

2. Data from only 9 of the 11 subjects who had completed the trial before the original article was submitted were included in the original article. These 9 subjects correspond to the 9 who had adipose tissue available for immunohistochemical analyses of p16^INK4A+^ cells and p21^CIP1+^ cells. Reasons the 2 subjects who had results for SA-βgal but not immunohistochemical results for p16^INK4A+^ and p21^CIP1+^ were not shown in Fig. 1 in our original article were: 1) for ease of comparison across subjects in Fig. 1 (each subject’s data were colour-coded) and 2) using the % transformation in the original article, decreases in SA-βgal were statistically significant whether the 9 or all 11 subjects were analyzed. However, using the statistical analyses in this Corrigendum for the 9 subjects, p=0.059, while for all 11 subjects, p=0.0226 (subject 10: 9.84 *vs*. 6.27 SA-βgal^+^ cells/nucleus at Days 0 and 14, respectively, and subject 11: 7.11 *vs*. 1.49).

3. We had to add 0.001 to take the log since there were 0’s in the data.

**Grouped Analyses**

The harmonic mean p-value (HMP) test, computed using the R package “harmonicmeanp”, was used to analyze the following pre-defined set of p-values for adipose tissue senescence-associated features: p16^INK4A+^ cells, SA-βgal^+^ cells, p21^CIP1+^ cells, adipocyte progenitor proliferation, CD68^+^ cells, and crown-like structures in the 9 subjects reported in the original article. The HMP is a method for performing a combined test of the null hypothesis that no p-value is significant (ref 3). For the 9 subjects in whom all 6 adipose tissue senescence-associated measures were available, the harmonic mean p=0.020. This also held if p16^INK4A+^ cells, SA-βgal^+^ cells, and p21^CIP1+^ cells are combined and analyzed by the HMP=0.036. Thus, there is an effect of D+Q on human adipose tissue senescence-associated measures.

**SASP Factors**

Analysis of SASP factors in the 9 of the 11 subjects whose data were in the original article.

| SASP factor^4^ | N | Assessment of Normality Assumption | | | Paired T-tests of log-transformed data |
| --- | --- | --- | --- | --- | --- |
|  |  | Shapiro Wilk test p-value | Skewness | Kurtosis | p-value |
| Composite score^5^ | 9 | 0.132 | -0.46 | -1.01 | **0.003** |
| IL-9 | 9 | 0.931 | -0.37 | -0.74 | 0.087 |
| IL-1a | 9 | 0.187 | -0.98 | -0.09 | 0.149 |
| IL-6 | 8^6^ | 0.063 | -0.76 | -1.17 | 0.152 |
| IL-2 | 9 | 0.290 | -0.57 | -0.60 | 0.14 |
| MMP-9 | 9 | 0.287 | -0.02 | -1.87 | 0.068 |
| MMP-12 | 8^6^ | 0.993 | -0.10 | -1.45 | **0.034** |
| MMP-2 | 9 | 0.623 | -0.27 | -0.52 | 0.092 |
| FGF-2 | 9 | 0.099 | -0.63 | -0.96 | 0.183 |
| IL-1RA | 9 | 0.971 | -0.03 | -0.76 | 0.199 |
| GM-CSF | 9 | 0.852 | -0.02 | -0.72 | 0.212 |

4. SASP factors are log-transformed and analyzed.

5. Composite Score is the average z-score of log-transformed values. See Appendix: Explicit mathematical expression of composite score.

6. Data missing for baseline or post treatment values for 1 subject.

**Skin**

**Log-Transformed Data**

| Skin parameter | N | Mean difference (SE) | Paired T-tests log-transformed |
| --- | --- | --- | --- |
| p16^INK4A+^ cells^7^ | 9 | -0.40 (0.32) | 0.164 |
| p21^CIP1+^ cells | 9 | -0.54 (0.29) | 0.092 |
| CD1a^+^ cells (control)^8^ | 9 | 0.50 (1.64) | 0.545 |

7. We had to add 0.001 to take the log since there was a 0 in the data.

8. CD1a^+^ cells (resident Langerhans cells; control) were not expected to be decreased by D+Q.

HMP was computed based on the p-values obtained from paired T-tests for log-transformed data for 2 epidermal parameters, p16^INK4A+^ and p21^CIP1+^ cells=0.118. Therefore, based only on the data from the 9 subjects who had skin assays of senescent cells in the original article, it is not possible to conclude with statistical confidence that D+Q had a significant effect on epidermal senescent cell burden.

**Raw Data**

In the interests of transparency, we included de-identified data from the original article in an appendix.

**Methods**

**Imaging Methods Clarification**

As reported in our article, adipose tissue and skin biopsy slides were scanned using a Motic Slide Scanner (Motic Company, China) and a 40× objective. In adipose tissue biopsies, images scanned for p16^INK4A^ and p21^CIP1^ were sliced (virtually) using Adobe Photoshop (Adobe Inc., San José, CA) to generate numerated fields that were 400 μm × 300 μm. Thirty fields were selected for analysis from each image using a random number generator, and when the random field chosen was blank or in a septal area of the tissue, the nearest field with adipose tissue was selected. p16^INK4A+^ and p21^CIP1+^ cells and adipocytes were counted by ImageJ. The total number of stained cells was calculated as a percent of the total number of adipocytes counted in all 30 fields. For macrophages, an Olympus BX43 light microscope (Olympus, Japan) with a 40× objective was used to image 10 random fields, which were manually counted for CD68^+^ macrophages, crown-like structures, and adipocytes using the software AMCounter (Biomedical Imaging Resource, Mayo Clinic, Rochester, MN). For skin biopsies, cells positive for p16^INK4A^, p21^CIP1^ (present in the basal layer), CD1a, and CD68 were counted and divided by the total length of the epidermis (mm) using ImageJ. All image analyses were conducted by observers who were blinded as to whether samples were pre- or post-treatment biopsies.

**Changes in Conclusions**

The main conclusion, that the senolytic combination, D+Q can decrease senescent cells in humans, is unchanged. Adipose tissue senescent cells decreased, as indicated by statistically significant decreases in p16^INK4A+^ cells, decreases in the macrophages that senescent cells can attract, anchor, and activate, decreases in adipose tissue crown-like structures, and statistically significant effects of D+Q on human adipose tissue demonstrated in grouped analyses of p16^INK4A+^, p21^CIP1+^, and SA β-gal^+^ cells, as well as of p16^INK4A+^ cells, p21^CIP1+^ cells, SA β-gal^+^ cells, CD68^+^ cells, crown-like structures, and adipose progenitors with limited replicative potential. Although we reported a statistically significant decrease in skin senescent cells in the 9 subjects whose skin data were reported in the original article, that conclusion did not hold up upon reanalysis of the non-transformed data. However, blood SASP factors were overall statistically significantly reduced after D+Q treatment, further supporting our finding of a senolytic effect of D+Q in humans.

Therefore, the overall conclusion of our article that D+Q can target senescent cells in humans holds upon re-analysis of the data, at least in adipose tissue and as reflected by a composite of blood SASP factors, but we have not shown this in skin here. That said, we emphasize, as stated in the conclusion of our article, members of the public should not use and physicians should not prescribe these agents to target senescent cells based on the available data. These agents should only be administered in the context of clinical trials with close monitoring. In our view, our conclusions need to be tested further as this and other clinical trials of senolytics progress. It is particularly important for other groups to conduct clinical trials with senolytics to confirm target engagement (senescent cell declines) in humans. If target engagement is further confirmed, clinical trials testing efficacy and effectiveness need to be completed before routine clinical use of senolytics can be considered.

**Acknowledgements**

We are grateful to the statistical teams helping with this report, including David Allison, Dean, Indiana University School of Public Health and his colleagues, Stephanie L. Dickinson and Keisuke Ejima; Elizabeth Atkinson, Assistant Professor of Biostatistics, Mayo Clinic; and Marc Lenburg, Professor, Section of Computational Biomedicine, Boston University. We are also grateful to our colleagues, the EBioMedicine editors and reviewers, and our readers for their insightful advice that guided the statistical analyses shown above. Each statistical team took responsibility for different components of this work.

**Appendix: Explicit mathematical expression of composite score**

$${Composite Score}_{i}=\sum_{j=1}^{m_{i}} \frac{z_{ij2}-z_{ij1}}{m_{i}}$$

, where $z_{ij1}$ and $z_{ij2}$ are z-scores of log-transformed values of plasma SASP factor *j* before and after treatment for patient *i*, respectively. $m_{i}$ is the number of observed factors (both before and after) for participant *i*.$z_{ijt}$ is calculated as follows:

$$z_{ijt}=\frac{\ln\left( x_{ijt} \right)-\mathrm{Mean}[{ln(x}_{j})]}{\mathrm{SD}[{ln(x}_{j})]}$$

, where $x_{ij1}$ and $x_{ij2}$ are raw values of plasma SASP factor *j* before and after treatment for patient *i*, $\ln\left( \cdot\right)$ is natural log, and $\mathrm{Mean}[{ln(x}_{j})]=\sum_{i=1}^{9} \sum_{t=1}^{2} \frac{\ln\left( x_{ijt} \right)}{n_{j1}+n_{j2}}$, and $\mathrm{SD}[{ln(x}_{j})]=\sqrt{\frac{\sum_{i=1}^{9} \sum_{t=1}^{2} \left\{ \ln\left( x_{ijt} \right)-\mathrm{Mean}[{ln(x}_{j})] \right\}^{2}}{n_{j1}+n_{j2}}}$. $n_{j1}$ and $n_{j2}$ are the number of participants for which plasma SASP factor *j* is available before and after D+Q treatment, respectively.

**References**

1. Minamino T, Orimo M, Shimizu I, Kunieda T, Yokoyama M, Ito T, Nojima A, Nabetani A, Oike Y, Matsubara H, Ishikawa F, Komuro I. A crucial role for adipose tissue p53 in the regulation of insulin resistance. *Nat Med.* 2009;15(9):1082-1087.

2. Xu M, Pirtskhalava T, Farr JN, Weigand BM, Palmer AK, Weivoda MM, Inman CL, Ogrodnik MB, Hachfeld CM, Fraser DG, Onken JL, Johnson KO, Verzosa GC, Langhi LGP, Weigl M, Giorgadze N, LeBrasseur NK, Miller JD, Jurk D, Singh RJ, Allison DB, Ejima K, Hubbard GB, Ikeno Y, Cubro H, Garovic VD, Hou X, Weroha SJ, Robbins PD, Niedernhofer LJ, Khosla S, Tchkonia T, Kirkland JL. Senolytics improve physical function and increase lifespan in old age. *Nat Med*. 2018;24(8):1246-1256.

3**.** Wilson DJ. The harmonic mean *p*-value for combining dependent tests. *Proc. Natl. Acad. Sci.* 2019;116(4):1195–1200.
